# NMR-Based Metabolic Profiling of Edible Olives—Determination of Quality Parameters

**DOI:** 10.3390/molecules25153339

**Published:** 2020-07-23

**Authors:** Stavros Beteinakis, Anastasia Papachristodoulou, Georgia Gogou, Sotirios Katsikis, Emmanuel Mikros, Maria Halabalaki

**Affiliations:** 1 Division of Pharmacognosy and Natural Products Chemistry, Department of Pharmacy, National and Kapodistrian University of Athens, Panepistimiopolis, Zografou, 15771 Athens, Greece; sbeteinakis@pharm.uoa.gr (S.B.); anpapac@pharm.uoa.gr (A.P.); gogo94go122@hotmail.com (G.G.); sotirisk@gmail.com (S.K.); 2 Laboratory of Cellular Immunology, Department of Microbiology, Hellenic Pasteur Institute, 127 Vas. Sofias av., 11521 Athens, Greece; 3 Division of Pharmaceutical Chemistry, Department of Pharmacy, National and Kapodistrian University of Athens, Panepistimiopolis, Zografou, 15771 Athens, Greece; mikros@pharm.uoa.gr

**Keywords:** *Olea europaea* L., edible olives, NMR, untargeted metabolomics, chemometrics, biomarker, STOCSY

## Abstract

Edible olive drupes (from *Olea europaea* L.) are a high-value food commodity with an increasing production trend over the past two decades. In an attempt to prevent fraud issues and ensure quality, the International Olive Council (IOC) issued guidelines for their sensory evaluation. However, certain varieties, geographical origins and processing parameters are omitted. The aim of the present study was the development of a method for the quality assessment of edible olives from the Konservolia, Kalamon and Chalkidikis cultivars from different areas of Greece processed with the Spanish or Greek method. A rapid NMR-based untargeted metabolic profiling method was developed along with multivariate analysis (MVA) and applied for the first time in edible olives’ analysis complemented by the aid of statistical total correlation spectroscopy (STOCSY). Specific biomarkers, related to the classification of olives based on different treatments, cultivars and geographical origin, were identified. STOCSY proved to be a valuable aid towards the assignment of biomarkers, a bottleneck in untargeted metabolomic approaches.

## 1. Introduction

The olive fruit is a drupe, oval in shape, produced by the olive tree (*Olea europaea* L.). Olives, based on cultivar, can either be used for the production of the well-known and high-valued olive oil or undergo processing and become an edible product. Consumption of edible olives worldwide has doubled since the year 2000, reaching a provisional 2.7 million tons in 2019, as more people gradually adopt the Mediterranean diet [1]. Greece, despite its small area, is estimated to account for 7% of the world production in 2020 [2].

Processing in the production of edible olives is essential to remove the bitter taste that makes them undesirable, which is due to certain phenolic compounds such as the secoiridoid glucoside, oleuropein [3]. Thus far, there are three main approaches with industrial scalability used for debittering olives, which include the Greek, Spanish and Californian processing methods. In short, in the Greek method, also known as natural or organic, harvested olives are brined in a sodium chloride solution and fermented with indigenous microbiota. In this case, the debittering occurs slowly and bitter compounds disappear due to enzymatic and microbial hydrolysis and diffusion to the brine solution. The entire procedure lasts from six to twelve months. For the Spanish or chemical method, harvested olives are subjected to lye treatment for several hours prior to brining, in order to forcefully hydrolyze bitter constituents. Washing and external addition of microbiota are essential for the removal of sodium hydroxide and lowering of the pH value. In total, the Spanish method usually takes only several days or weeks to complete, and in extreme cases, months. The Californian method, another chemical method introduced recently, and the least used of the three, can utilize both brined (for a few months) and non-brined green harvested olives, which will undergo several lye treatments to remove the bitter taste. Olives from the above methods have to undergo pasteurization prior to consumption [4,5].

Olives consist of approximately 50–60% water and 20% fat, while the remaining part is comprised of secondary metabolites, sugars, cellulose and nitrogenous compounds [6]. In recent years, much attention has been given to certain constituents of olives which are characterized by important biological and pharmacological activities, and are also implicated in quality determination [7,8,9,10,11,12]. In general, small molecules found in olive drupes belong to one of the following chemical classes based on structure: phenolic acid derivatives (i.e., caffeic acid), phenylethanoid derivatives (i.e., hydroxytyrosol, tyrosol), flavonoids (i.e., luteolin), lignans (i.e., pinoresinol), secoiridoids (i.e., elenolic acid), tocopherols and triterpenic acids (i.e., maslinic acid) [13].

It is important to note that the composition of edible olives regarding secondary metabolites can be affected by both endogenous (olive variety) and exogenous (storage, irrigation, processing, geographical origin) factors [14]. These parameters are directly associated with the quality and authentication of edible olives [15,16]. As usually happens for other appreciable food products, the quality requirements arise along with production rates; in order to prevent frauds, the International Olive Council (IOC) issued in 2011 a directive imposing a mandatory organoleptic assessment performed by a trained sensory panel in order to classify edible olives. Negative attributes, like musty and rancid, descriptive gustatory attributes, such as bitter or acid and kinesthetic sensations, hardness, crunchiness and fibrousness, are used to classify table olives according to the defect predominantly perceived (DPP). The highest-ranking class is labeled as extra or fancy, followed by first (or 1st), choice or select and second (or 2nd) or standard for the second and third classes, respectively. The fourth and final class is comprised of olives that should not be sold as table olives [17].

However, geographical origin, processing and cultivar fraud issues require additional analyses to be determined. For instance, the significantly less time needed for the Spanish processing method could make it a preferable option against the Greek one, even in black olives. The drastic decrease in the content of nutritional compounds of the processed product is nevertheless not in the best interest of the consumer [16,18,19,20]. On the other hand, variety and geographical origin determination is a mandatory prerequisite in order for a product to be labeled as protected designation of origin (PDO) or protected geographical indication (PGI), leading to significant added value [21].

In most cases, when quality aspects are implicated, analytical methods and techniques are employed to resolve given issues by suggesting certain methodologies or quality markers [22,23]. Generally in foods and food stuffs, gas chromatography hyphenated to flame ionization detectors (GC-FID) or mass spectrometers (GC-MS), or liquid chromatography coupled with ultraviolet detector (LC-UV) or mass spectrometers (LC-MS) are commonly used as means of mainly quantitative or metabolic profiling methods [24,25,26]. However, available analytical data for processed olives suitable for oral consumption are minimal concerning quality and authentication characteristics, such as geographical origin, variety, storage, processing style determination, etc.

Aside from the aforementioned established method of sensory analysis, GC-MS with multivariate analysis (MVA) has been used to determine spoilage issues, where volatile organic derivatives are identified as biomarkers [27], or to define cultivar and processing types using fatty acid composition as the differentiation factor [28]. Fibers, sugars, organic acids and minerals have also been determined as markers for cultivar and processing type discrimination by methods such as atomic absorption spectroscopy (AAS), flame photometry (FP) or high-performance liquid chromatography (HPLC) [29,30]. Furthermore, visible-near infrared spectroscopy (Vis-NIRS) has been used to predict olive color successfully, failing though in the prediction of firmness [31]. Finally, as far as olive drupes from Greece are concerned, antioxidant capacity and total phenol content (TPC) are among the main subjects studied so far [32,33,34], while phenolic [32,34], organoleptic and microbiological profiles [35,36] have also been investigated. Individual molecules have been studied to some extent as well, mostly focusing on quantification aspects [37,38]. Nuclear magnetic resonance (NMR) spectroscopy in combination with chemometrics has been successfully used in the authentication of a wide range of food commodities, like olive oil, meat, honey and saffron [23,39,40,41]. To our knowledge, no application has been reported using the NMR-based metabolic profiling tool for table olives, while other analytical methods represent a handful of studies.

Furthermore, regardless of the analytical method used, a critical bottleneck in metabolic profiling methods is the identification of relevant compounds that could serve as quality markers. Indeed, one of the most critical steps in metabolomics approaches involving food (foodomics) and natural products in general is the identification of relevant biomarkers, mainly due to the lack of databases and the frequent absence of reference standards, as well as the complexity, high variability and unexpected nature of natural matrices. Thus, statistical techniques and tools have been suggested to assist with this issue [42]. Among these lines, statistical total correlation spectroscopy (STOCSY) has been proposed and employed mainly to human or animal samples (e.g., plasma, urine). STOCSY is applied on NMR datasets and takes advantage of the multi-co-linearity of the variables’ intensity in a set of spectra to generate pseudo-NMR spectra which display the correlation of intensities of the various peaks across the whole sample [43]. STOCSY correlations between certain peaks indicate the possibility of belonging to the same compound or to compounds of the same origin pathway. To our knowledge, STOCSY has been, up till now, used only in the study of some medicinal plants like *Paeonia mascula* [44], *Cinchona* sp. [45], *Isatis tinctorial* [46] and *Keetia* sp. [47], and a couple of other cases [48,49,50], while there is a recent study by Kew et al. involving Scotch whisky [51].

Consequently, the objective of this study was the development of an NMR-based metabolic profiling method to map edible olives, but also to confront mislabeling and fraud. Chemometrics were employed for the identification of certain marker compounds, which were verified via STOCSY, a tool used for the first time in edible olive analysis.

## 2. Results and Discussion

### 2.1. Sample Collection

Edible or table olives constitute, directly after olive oil, valuable products mainly for Mediterranean countries, such as Greece. Nevertheless, there are limited studies that report the mapping of Greek edible olives or investigate quality aspects and authentication issues. In Greece, there are approximately fifteen varieties, not all intended for table use, which mainly originate from specific areas of the territory. Primary edible varieties are Kalamon, Chalkidikis, Konservolia and Throumbolia [52,53]. Thus, the first step of the present study was to organize a systematic collection of edible olives samples reflecting a varietal and geographical representation. Moreover, samples from the two most common processing methods, Greek and Spanish, were included (Table 1). Specifically, edible olives from three geographical areas of Greece, i.e., Makedonia, Sterea Ellada and Peloponnese, and several subregions, like Kavala, Fthiotida and Aitoloakarnania, corresponding to the north, center and south parts of Greece, were collected.

All samples were stored, preserved and extracted under the same conditions prior to analysis in order to minimize any induced variability. Furthermore, complete information accompanying each sample (metadata) was registered (Table S1).

### 2.2. Acquisition, Data Processing and Multivariate Analysis

Over the last several years, NMR is increasingly being introduced in the quality control of foods, offering important advantages, i.e., speed, reproducibility and simplicity [54,55]. To that end, NMR metabolic profiling was selected in the current study in order to analyze the 60 collected samples following an *in-house* developed protocol. Considerable attention was given to parameters such as the selection of the drying process, extraction solvents and yield, required quantity for analysis, internal standard (IS) and deuterated solvent used (data not shown).

Samples were dissolved in methanol-*d*_4_ with Hexamethyldisiloxane (HMDSO) as an IS and a line-shape indicator for the recorded spectra. A total of 60 spectra were acquired using a simple zg pulse sequence.

**Figure 1:**
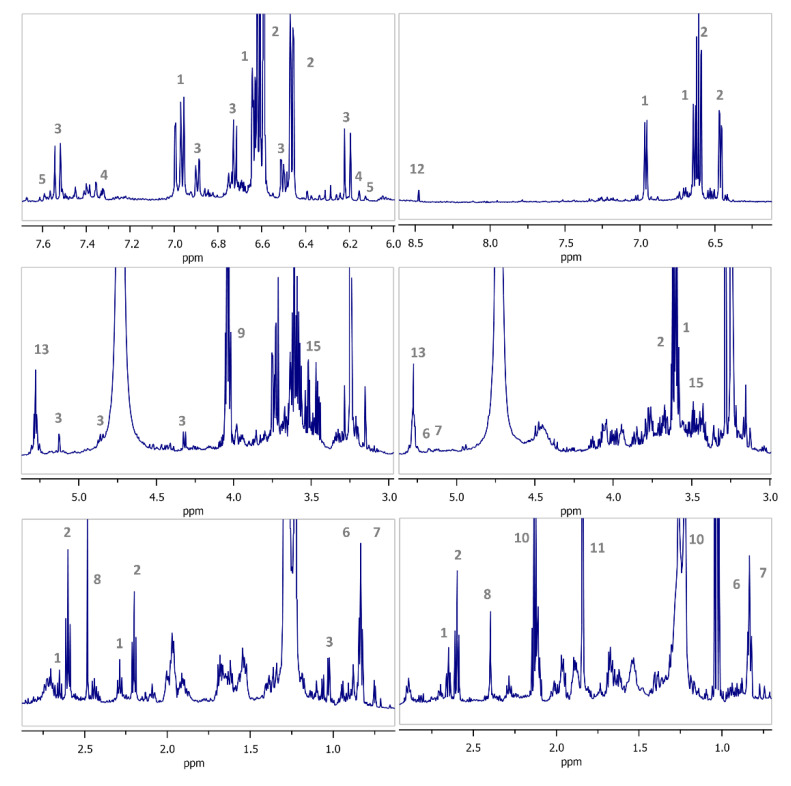
Annotated representative 1D ^1^H NMR spectra from two olive varieties (Kalamon and Chalkidikis) from two processing methods and different geographical origin. Tyrosol (**Τyr**), **1**; Hydroxytyrosοl (**HT**), **2**; Verbascoside (**Ver**), **3**; Luteolin (**Lut**), **4**; Quercetin (**Quer**), **5**; Maslinic Acid (**MA**), **6**; Oleanolic Acid (**OA**), **7**; Succinic Acid (**SA**), **8**; Lactic Acid (**LA**), **9**; Propionic Acid (**PA**), **10**; Acetic Acid (**AA**), **11**; Formic Acid (**FA**), **12**; Triacylglycerols (**TAGs**), **13**; Linoleic Acid (**Lin**), **14**; Glycerol **(G**), **15**.

Representative spectra from two varieties (different geographical origin and processing method) are given in Figure 1 and signals of identified compounds are annotated, providing a coarse idea of the samples’ nature. Full peak assignment is given in Table 2. Qualitative differences are evident between the two samples even with visual inspection. Furthermore, quantitative variations of the peaks corresponding to fluctuations in metabolites’ levels are also observed. For instance, phenolics’ signals which resonate downfield seem to vary considerably between the two spectra, along with signals upfield, indicative of aliphatic chains. Chemical shifts’ differentiation is evident between 3 and 5 ppm, where commonly protons of double/triple bonds, heterocyclic and oxygenated systems resonate, i.e., carbohydrates and polyols.

Due to pH variances, minor shifts were observed in certain peaks running through the spectra. To that end, the icoshift tool in the MATLAB suite was applied with specifically chosen intervals for the alignment of all spectra. IS normalization was applied to eliminate any induced variability originating from sample preparation and acquisition of the spectra. The datasheet was imported into SIMCA v. 14.1 to perform MVA. Geographical origin, tree cultivar and processing type were the studied classes characterizing the observations (samples).

In order to form the first visual opinion of the data, detect any inherent pattern within them and determine possible outliers, principle component analysis (PCA) was performed. In the current study, Pareto scaling along with logarithmic transformation were used to construct the statistical models, as weight adjustment was mandatory for major metabolites. As a next step, partial least squares discriminant analysis (PLS-DA) was performed using the same datasheet of variables. Finally, orthogonal partial least squares projections to latent structures with discriminant analysis (OPLS-DA) models were built by taking groups in pairs, in order to identify certain markers responsible for their differentiation. Outliers were not detected in any built model.

#### Geographical Origin, Olive Cultivar, Processing Procedure

**Figure 2:**
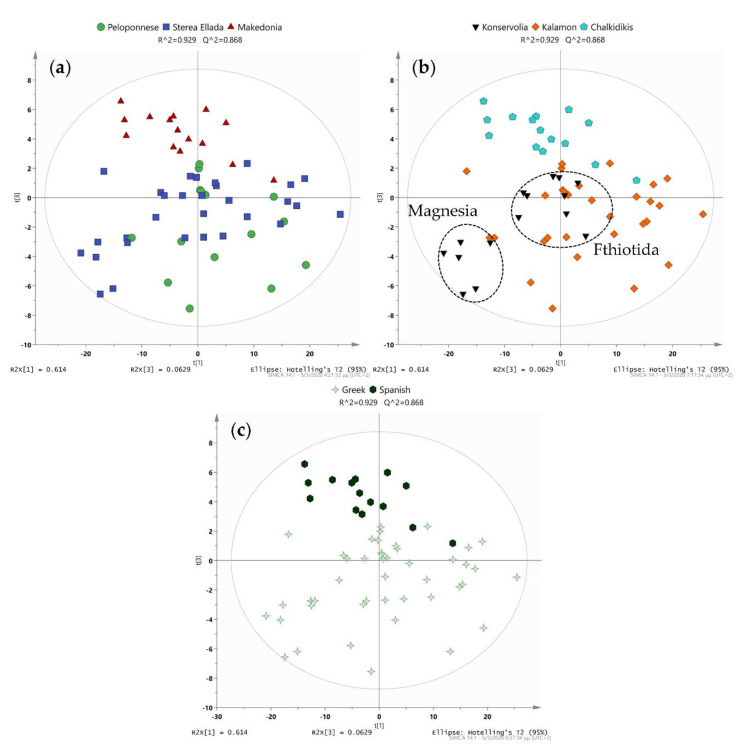
Statistical analysis of all parameters. (**a**) Principal component analysis (PCA) scores plot showing the forming pattern between regions; (**b**) PCA scores plot of the variety parameter. Two clusters are observed within Konservolia; (**c**) PCA scores plot examining processing. Distinct separation is observed.

Three regions with the highest production in Greece—Peloponnese (south), Sterea Ellada (center) and Makedonia (north)—were used for sample collection to study the impact that geographical origin might have on the samples’ nature. Firstly, in the PCA model (Figure 2), four first components accounted for more than 80% cumulative variation in the data with the most significant separation observed in PC1 and PC2. A distinct classification was evident in samples from Makedonia, in the northern part of Greece, while Peloponnese and Sterea Ellada were not separated, despite the evident trend. This observation is justified as Peloponnese and Sterea Ellada share more common climatological and soil conditions compared with Makedonia. For the construction of the PLS-DA model, three components were used. As expected, a clearer separation than in PCA was observed, with samples from Makedonia forming a distinct cluster. Certain samples from Sterea Ellada and Peloponnese were, however, still intertwined, showing once again the relevance between these regions. The model was validated with permutation tests for each of the three regions (500 permutations used, Figure S2), showing that the separation was due to alterations amongst samples and the models were not overfit.

Finally, three OPLS-DA models were built by taking regions in pairs with the end-goal being the identification of compounds distinct to each region. R^2^ and Q^2^ values verified the goodness of fit and predictivity of the models, with the issue between Sterea Ellada and Peloponnese revealing once more the similarities between these samples. To facilitate model interpretation, we constructed the respective S-plot to each OPLS-DA model, that provides visualization of its predictive component loading. However, the use of variable importance for the projection (VIP) values of the features of each OPLS-DA loadings plot was what actually led to the selection of the most relevant features. Features with a VIP > 1 and a larger effect in class discrimination were selected (Table S2) and further evaluated [44].

Moving on to cultivar, three of the four most common olive cultivars of Greece were included in the present study, Kalamon, Konservolia and Chalkidikis [53]. Similar to geographical origin, PCA was initially performed to investigate possible patterns between cultivars. The first two components of the model accounted for more than 70% cumulative variation in the data (Figure 2). Expectably, Chalkidikis was definitely clustering, whereas in Kalamon and Konservolia, clustering was accompanied by strong overlapping in some samples. Interestingly, two subgroups were observed in the Konservolia samples, which with close inspection proved to be due to the different geographical origins, i.e., Magnesia and Fthiotida. This observation was also reported in the OPLS-DA plots, constructed to compare cultivars in pairs (Figure S4). Clear grouping and separation between Magnesia and Fthiotida were once again verified, while subgroups related to geographical origin were not observed in either Chalkidikis or Kalamon cultivars. Our findings suggest that geographical origin seems to influence significantly the phenolic composition of olives, even more than cultivar, at least in the case of Konservolia.

Similar to the case of geographical origin, further validation was performed with permutation tests for each cultivar, indicating the statistical significance of the aforementioned values (Figure S3). An identical workflow was also adopted for the discovery of the most relevant features responsible for differentiation among cultivars. OPLS-DA models, built with two classes per model, showed Q^2^ and R^2^ values very close to 1 (Figure S4), verifying their validity. VIP tables extracted from each model were further analyzed in order to identify marker compounds (Table S2).

Concerning the processing procedure, edible olives processed with either the Greek or the Spanish method are represented in this study. As mentioned before, the Greek method is a more natural way of processing mainly purple or black olives that is comprised of brining and fermentation, whereas the Spanish includes an initial additional step of lye treatment and is used mostly for green olives. During lye treatment, olives are placed in large tanks containing sodium hydroxide solution (1.3–2.6% *w/v*), where they remain for 4–15 h until the solution penetrates the skin and approaches the pit, pushing the mutation of the chemical profile in a more forceful manner [16,18,53].

Yet the PCA model highlights the remarkable discrimination based on different processing methods (Figure 2). This observation was further reinforced with the respective PLS-DA model (Figure S5). Q^2^ and R^2^ values, along with the carried-out permutation tests, verified the reliability of the model (Figure S5). The respective OPLS-DA model was once again constructed as a means to an end, with the final step being the S-plot and the extraction of a VIP list for the mining of biomarkers responsible for the observed clustering (Table S2). With that being said, the model built with one predictive and three orthogonal components was checked for its validity with Q^2^ and R^2^ both above 0.75.

### 2.3. Statistical Total Correlation Spectroscopy (STOCSY) and Biomarker Identification

As already mentioned, a really critical step in untargeted metabolic profiling approaches is the mining of relevant features followed by their translation into compounds, which could function as possible markers. Nevertheless, the identification process which commonly involves the dereplication of known compounds is a challenging task [42]. Thus, in the current study a step-by-step methodology was followed to initially reveal significant loadings using MVA, while STOCSY was, in turn, applied for the identification of relevant markers. Chemical shifts from the generated VIP lists (VIP > 1) of the OPLS-DA models were used as “driving peaks” in the STOCSY experiments. STOCSY plots derive from the correlation between the selected statistically significant loadings or peaks and all other NMR data points to produce the respective pseudo-spectra. Removal of solvents’ chemical shifts (methanol-*d*4, methanol, water) was part of the experimental protocol.

**Figure 3:**
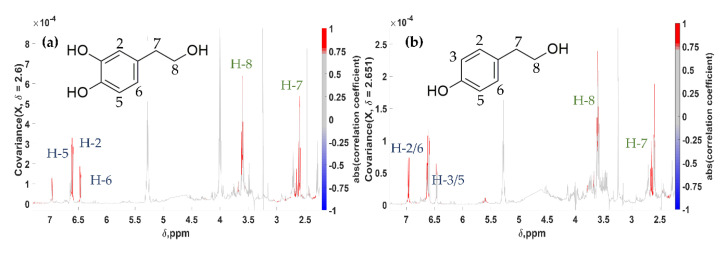
Statistical total correlation spectroscopy (STOCSY) 1D pseudo-NMR spectra. Correlation coefficients to the other signals in the median edible olive NMR spectrum are color-encoded. (**a**) **HT**: “driving peak” was at 2.600 ppm; (**b**) **Tyr**: “driving peak” was at 2.651 ppm; complete assignment in Table 2.

In more detail, the resonance at 2.600 ppm (VIP No 16 in Chalkidikis vs. Konservolia and No 41 in Kalamon vs. Konservolia, Table S2) was used as a driving peak, revealing a set of others indicative for the compound Hydroxytyrosol (**HT**) (Figure 3a). Using 2.651 ppm as a driving peak, another molecule, Tyrosol (**Tyr**), was identified (Figure 3b). **HT** and **Tyr** are characteristic phenylethanoids present in both fresh and processed olives, as well as in olive oil and olive by-products [13,67,68].

At the covariance spectra, all peaks corresponding to the protons of both molecules are evident, i.e., the ABX and A2B2 spin systems for **HT** and **Tyr**, respectively, and the two methylene groups of the side-chain for each compound. The two molecules present strong structural similarities, with **HT** being a hydroxylated derivative of **Tyr**, which is reflected in the pseudo-spectra as well. It is important to note that both multiplicity and peak integration are also apparent and in accordance with their reference ^1^H NMR spectra (Table 2). Both being major phenolics in table olives with an estimated range of 110–980 for **HT** and 40–135 for **Tyr** expressed in mg/kg of olive flesh according to Boskou et al. [16], they are clearly projected with no interference from peaks belonging to other compounds (noise or artefact peaks) and a correlation coefficient > 0.75. Moreover, no negative peaks (correlation coefficient < 0.00) were observed showing any anti-correlation with **Tyr** and **HT**.

**Figure 4:**
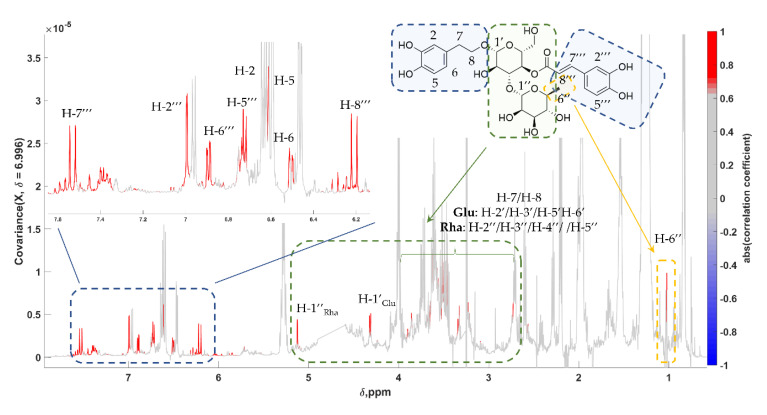
STOCSY 1D pseudo-NMR spectrum of **Ver**. Correlation coefficients to the other signals in the median edible olive NMR spectrum are color-encoded: “driving peak” was at 6.994 ppm. Zoom-in of the aromatic region is also presented.

The same workflow was followed for verbascoside or acteoside, a caffeoyl phenylethanoid glycoside. It is a structurally complex molecule, which is composed of a disaccharide formed by a glucose and a rhamnose unit, to which caffeic acid and **HT** are attached with an ester and an ether bond, respectively. Verbascoside (**Ver**) is considered a minor compound in edible olives [69], found also in olive paste [70]. However, the STOCSY tool made possible the unveiling of almost all peaks corresponding to resonances of this compound (Figure 4). As expected, the pseudo-spectrum was more crowded, especially in the sugars’ region, without nevertheless affecting the dereplication process and identification confidence.

**Figure 5:**
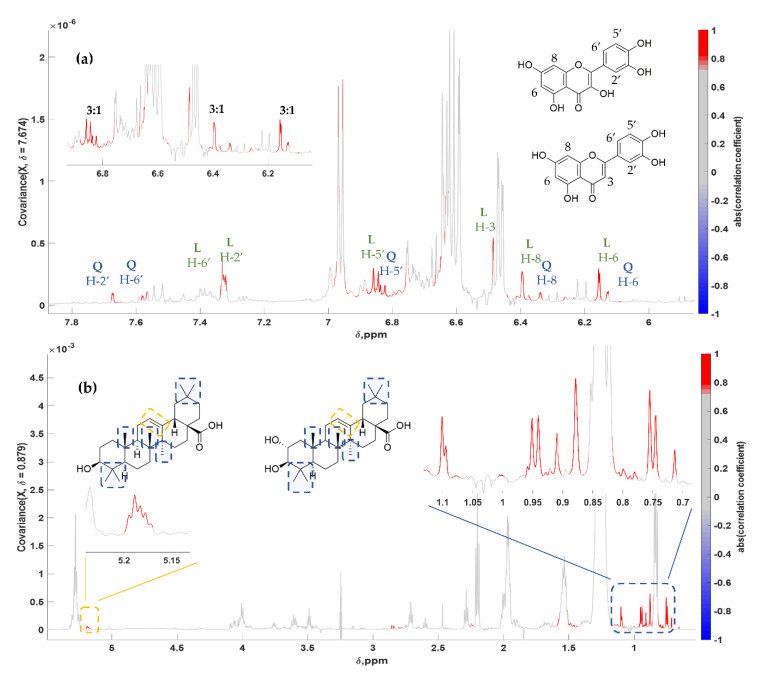
STOCSY 1D pseudo-NMR spectra. Correlation coefficients to the other signals in the median edible olive NMR spectrum are color-encoded. (**a**) **Lut** (bottom) and **Quer** (top): “driving peak” was at 7.674 ppm, peak integration L/Q 3:1 approximately; (**b**) **MA** (right) and **OA** (left): “driving peak” was at 0.879 ppm.

Similar to **Ver**, two flavonoids, Quercetin (**Quer**) and Luteolin (**Lut**), were also identified. Both compounds are also minor constituents of table olives with **Quer** present in lower concentrations compared with **Lut**, after hydrolysis of the respective glucosides due to processing [71]. Further, in this case, indicative correlation spectra for both compounds were derived. Interestingly, comparing the well resolved respective peaks of the compounds (L/Q H-6, H-8, H-5′), it was even possible to estimate their relative abundance (3:1 approximately) (Figure 5a). This is another strong point of STOCSY, as the quantitative potentials of NMR could be exploited and different compounds with proportional fluctuation could be identified.

Triterpenic acids are structurally complex constituents of table olives with the more characteristic being Oleanolic Acid (**OA**) and Maslinic Acid (**MA**). A wide range of biological activities has been linked with them over time, like antitumor, antidiabetic, antioxidant, cardioprotective, antiparasitic and even neuroprotective [10,72]. Both are pentacyclic triterpenic acids, with **MA** being a hydroxylated derivative of **OA** without any other structural differences. It is worth mentioning that in the generated pseudo-spectrum, all methyl protons, which resonate between 0.7 and 1.2 ppm, were clearly revealed, showing in this case too the average relative concentration of these compounds in the analyzed samples (Figure 5b, Table 2). Due to their proportional fluctuation in their concentration levels among samples, no distinct pseudo-spectra could be constructed for these compounds, resulting in a single one, where the correlation coefficient was over 0.80 for both with the same driving peak.

**Figure 6:**
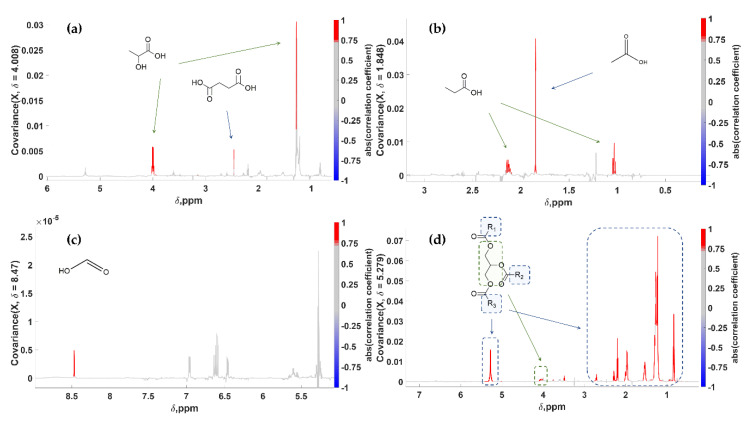
STOCSY 1D pseudo-NMR spectra. Correlation coefficients to the other signals in the median edible olive NMR spectrum are color-encoded. (**a**) **LA** and **SA**: “driving peak” was at 4.008 ppm; (**b**) **PA** and **AA**: “driving peak” was at 1.848 ppm; (**c**) **FA**: “driving peak” was at 8.470 ppm; (**d**) **TAGs**: “driving peak” was at 5.279 ppm.

Short-chain fatty acids (**SCFA**) constitute typical metabolites of processed table olives. Specifically, in the samples at hand, Formic (**FA**), Acetic (**AA**), Propionic (**PA**) and Lactic (**LA**) Acids accompanied by Succinic Acid (**SA**) had their ^1^H NMR spectra clearly projected through STOCSY. Their correlation spectra are truly indicative, enabling their identification with high confidence (Figure 6, Table 2). As depicted, **SA** with **LA**, as well as **PA** with **AA** were projected in pairs. This observation implies a bio-synthetic association of the detected metabolites and can be exploited further when STOCSY is employed aside from its identification potential. Finally, a pseudo-spectrum indicative for Triacylglycerols (**TAGs**) was generated using the peak at 5.27 ppm as a driver. Mainly **TAGs,** but also diacylglycerols (**DAGs**) and free fatty acids (**FFAs**) are highly abundant in drupes [73,74]. As a result, the generated pseudo-spectrum was highly informative and free of any artefacts. Overall, this VIP-based untargeted approach, integrating STOCSY as a dereplication tool, led to nine generated pseudo-NMR spectra and thirteen statistically significant compounds.

In parallel, a targeted approach, based on the literature search, led to the identification of linoleic acid and glycerol (Table 2). An extensive search, that would reveal compounds that have been discovered so far in edible olives, was carried out. Limited studies exist to our knowledge, that investigate processed olives and their phenolic composition. Indeed, most involve fresh olives [34,75,76,77], while there were others that used olives intended only for oil production [70,78]. To this date, the content of processed edible olives has been mainly investigated by HPLC-DAD [15,37,73,74,75,76,77] and MS [32,56,57,58,59,78], with NMR coming in a resounding third place [60]. It is important to note that minor deviations compared with the literature were observed, in some cases, in chemical shifts due to pH or calibration mismatches. Therefore, 2D spectra (JRES, HSQC and HMBC) were recorded in specific samples to ensure structure elucidation confidence (*data not shown*).

### 2.4. Quality and Authentication Assessment

Fraudulent practices in edible olives are numerous with the common denominator being adulteration and mislabeling. Organoleptic assessment suggested by the IOC, even though it provides a somewhat quality classification based on sensory attributes, cannot identify differences in other quality parameters, such as geographical origin, cultivar or processing method. Thus, the identification of specific markers or patterns indicative of these traits would be of utmost importance in the quality assessment and authentication of edible olives. In the current study, special attention has been given to the identification of specific measurable chemical markers indicative of analyzed cultivars or geographical origins, as well as processing methods supporting Greek PDO and PGI products.

Thus, following the initial experimental design, our team, in turn, focused on the identified and statistically significant compounds by monitoring their relevant concentration in the different classes. **HT** and **Tyr**, the two main phenylethanoids found in processed edible olives, have been extensively investigated for their biological and pharmacological properties [79,80]. Worth highlighting is the fact that, in a recent EFSA claim, **HT** and its derivatives were correlated with a protective effect over blood lipids from oxidation resulting in cardio-protection for the consumer. The health claim concerns olive oils with certain concentration levels of these compounds [81]. Therefore, use of the claim is allowed on labels of bottled olive oils, but fails to include table olives, another rich source of these compounds.

**Figure 7:**
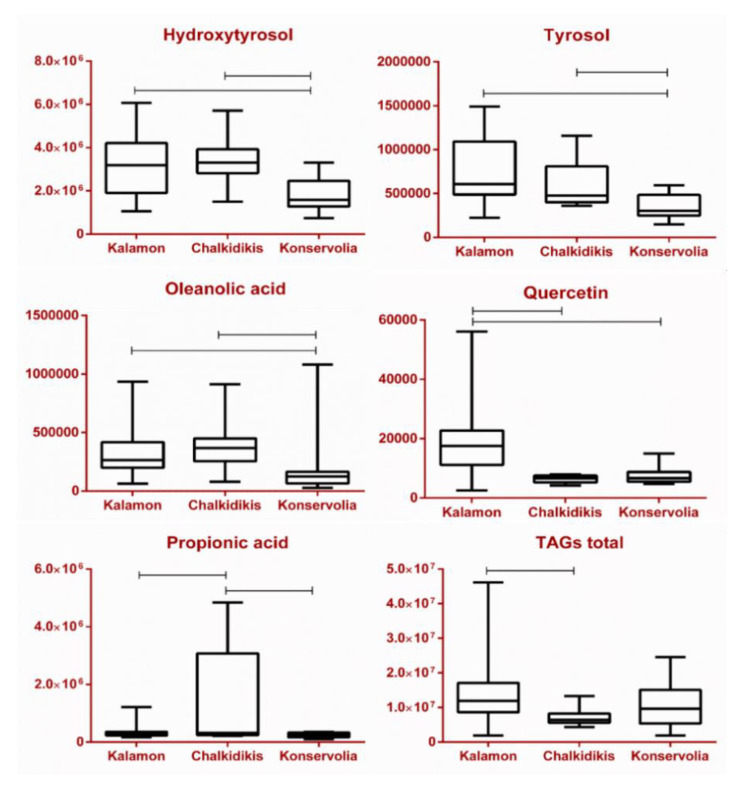
Box plots of a selection of statistically significant markers in the parameter of variety. Specifically, **HT**, **Tyr**, **OA**, **Quer PA**, and total **TAGs** are depicted (vertical axis expressed in absolute intensity).

Based on the box plots presented below, Kalamon and Chalkidikis cultivars (Figure 7) are richer in both **HT** and **Tyr**, compared with Konservolia, the other black olive variety of the present study, agreeing with the literature [15,16,18]. This is in accordance with their relative concentration when geographical origin is taken into account. Sterea Ellada shows the lowest abundance in both compounds compared with Makedonia and Peloponnese (Figure S7). Recent studies show a probable effect of the Greek method towards higher concentrations of **HT**, whereas no effect was reported in the levels of **Tyr**. The respective t-test applied in this study reveals the possible significance of **HT** with a p value of 0.06, while the respective value for **Tyr** was 0.82, showing no statistical significance (Table S3).

**Figure 8:**
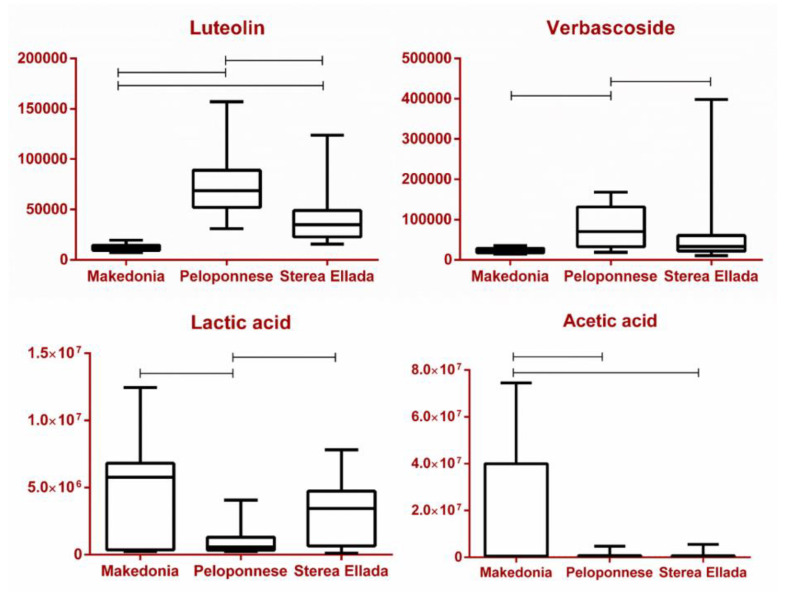
Box plots of a selection of statistically significant markers in the parameter of geographical origin. Specifically, **Lut**, **Ver, LA** and **AA** are depicted (vertical axis expressed in absolute intensity).

A similar trend regarding cultivars is observed with **Ver** [15]. During processing, **Ver** is hydrolyzed, having as a result generally low levels in table olives, however detectable with NMR. In the current study, its detection was possible almost only in samples from the Kalamon cultivar (Peloponnese and Sterea Ellada). Moreover, **Ver** was detected in Peloponnese samples and in lower levels in Sterea Ellada, while being absent in samples from Makedonia (Figure 8 and Figure S8). This observation might imply that **Ver** is a characteristic marker of the Kalamon variety with geographical origin not affecting its concentration considerably. Regarding the processing method, while the respective p value of the t-test implies statistical significance for **Ver**, no solid conclusion could be extracted since Chalkidikis is the only variety having undergone Spanish processing. These observations are in accordance with Sahan et al., who noted a direct correlation between the processing method and the levels of **Ver** in table olives [20].

An analogous observation was made in the case of **Lut** and **Quer**, the two flavonols abundant in olive parts and products. Both were found in considerably higher amounts in Peloponnese and the Kalamon cultivar as opposed to Makedonia and the Chalkidikis cultivar. Typically, they are found in different glycosidic forms in fresh olives, but they are hydrolyzed during their treatment towards becoming edible. Similarly to **Ver**, it seems that both compounds are markers of the Kalamon cultivar and secondarily of Konservolia, while they do not seem to be influenced by geographical origin. Regarding processing, the step of lye treatment of the Spanish method seems to severely increase diffusion and/or hydrolysis of **Lut** and **Quer** glycosides, therefore the aglycons are found solely in Greek-style black olives (Kalamon and Konservolia), as not even traces were found in samples from Makedonia and the Chalkidikis cultivar (Figure 7, Figure 8 and Figure S8) [16,18].

Triterpenic acids are also characteristic compounds found in edible olives, predominantly **MA** and **OA**, found in olive oil, olive leaves and olive mill wastewaters [55]. Both compounds were found in lower concentrations in the Konservolia cultivar compared with Chalkidikis and Kalamon (Figure 8, Figure S7), as well as in Sterea Ellada compared with the other regions. This observation comes in contradiction with previous studies, where Konservolia was presented as superior compared with Kalamon when it came to **MA** [15]. Their concentration seems to differ amongst cultivars, but does not seem to be affected by processing according to the respective t-test (Table S3), which once again comes in contrast with the literature [37]. Moreover, **MA** seems to constantly outweigh **OA** in terms of concentration, a finding also confirmed by the present study [15,37,66].

As mentioned above, **TAGs** constitute 20% of the total weight of an olive drupe. Despite their lipophilic nature, they are also present in the samples to some extent. Studies have shown that the processing method might have an impact on the levels of **TAGs**. In particular, the sodium hydroxide solution applied during the Spanish method considerably increases diffusion of all **TAG** derivatives in the solution, as well as accelerates the oxidation of the unsaturated ones [19,20]. Our findings verified the statistical significance of total **TAGs** in the discrimination between Spanish and Greek (t-test, Table S3). This comes in agreement with the respective box plot among cultivars (Figure 8), as Chalkidikis contains noticeably lower concentrations in comparison with Konservolia and Kalamon, with the former having undergone the Spanish method and the latter two having undergone the Greek one.

**Figure 9:**
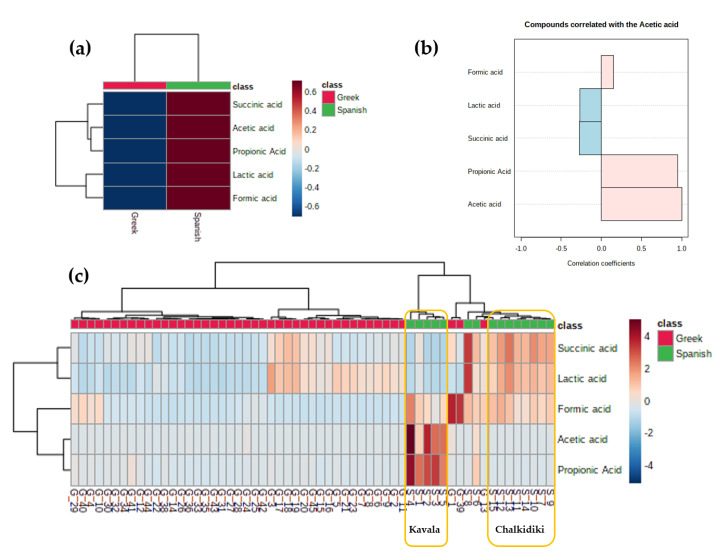
(**a**). Heat map plot for the selected short-chain fatty acids (**SCFAs**) considering only groups’ averages; (**b**). correlation analysis plot of acetic acid; (**c**). heat map visualization of **SCFAs** across the samples. Two classes, Greek (red) and Spanish (green), were considered. x axis: observations (samples), y axis: SCFAs loadings. Color-coded according to their correlation coefficient.

An interesting class of compounds detected in the analyzed samples was **SCFAs,** specifically **FA**, **AA**, **PA** and **LA** and dicarboxylic acid **SA**. It is well documented that these compounds are produced during the fermentation process mainly from *Lactobacillus* species. Moreover, higher concentrations have been reported in Spanish- rather than in Greek-type olives [29,82,83]. Based on our data, considering geographical origin and cultivar, a trend was observed. To elaborate, **LA** and **SA** presented the same pattern, as they were found in descending levels in Makedonia > Sterea Ellada > Peloponnese, as well as in Chalkidikis > Konservolia > Kalamon. Similarly, **PA** and **AA** were found solely in Makedonia and the Chalkidikis cultivar, while **FA** was found additionally in Peloponnese and the Kalamon cultivar (Figure 7, Figure 8, Figures S7 and S8). Furthermore, all five metabolites were found to be significant markers in favor of the Spanish method (Table S3). This could be easily observed in the respective heat map taking into consideration only groups’ averages (Figure 9a). Interestingly, **FA**, **AA** and **PA** seem to characterize olives produced with the Spanish method compared with the Greek one, in which they are practically absent. On the other hand, **LA** and **SA** are detected in samples from both methods with an obvious predominance in chemically processed samples (Figure 7, Figure 8, Figures S7 and S8). Indeed, in the correlation coefficients’’ plot (Figure 9b), the association between **PA**, **AA** and **FA** is evident, when compared with **LA** and **SA**. To that end, the STOCSY tool, as stated previously, had already implied a bio-synthetic association of **PA** with **AA** and **LA** and **SA**, as they appeared in pairs in the generated pseudo-spectra.

To achieve a better insight, intra-group correlations were investigated. As displayed in the heat map of Figure 9c, discrimination between the two groups (Greek vs. Spanish) based on the **SCFAs** is apparent. Even if all monitored metabolites are markers for the Spanish processing method, their contribution varies. Indeed, samples which are characterized by high levels of **LA** and **SA** are low in **AA** and **PA**, and vice versa. **FA** on the other hand seems to follow a not so clear and intermediate pattern. However, with closer inspection of the metadata, it seems that geographical origin is also important when a marker is to be chosen. Even if all samples processed with the Spanish method are of the same cultivar and geographical origin, subregion seems to play a crucial role. In particular, **AA** and **PA** appear to be better markers for the subregion of Kavala, while **LA**, **SA** and **FA** for the subregion of Chalkidiki. Nevertheless, it has been reported that the presence of **PA** and **AA** with the simultaneous absence or significant decrease in **LA** in certain Spanish-type samples were proven to be an indicator of decomposition in fermented products [4,81]. Thus, further studies are needed in order to elucidate the role of geographical origin and/or spoilage in the observed **SCFAs** and **SA** trends in processed table olives.

## 3. Materials and Methods

### 3.1. Collection of Samples

Edible olives, belonging to different varieties, color and processing styles, were handpicked and provided by producers from various regions around Greece. In the present study, 60 samples were totally analyzed. All samples were stored in their brines in dark conditions and under room temperature (25 °C).

### 3.2. Sample Preparation

#### 3.2.1. Lyophilization

A total of 10 olives per sample were picked randomly from the ones provided by the producers. They were lyophilized until the total loss of water content. Afterwards, pits were removed and the flesh was turned into powder manually.

#### 3.2.2. Extraction Protocol

Official extraction protocols for edible olive analysis have yet to be issued by the IOC. Consequently, the respective official one for olive oil analysis was used in this study and developed to match the different nature of the samples [84]. Specifically, 0.3 g of homogenized olive powder was accurately weighed in a 50 mL tube container (falcon type, ISOLAB Laborgeräte GmbH, Germany) and 30 mL of methanol/water: 80/20 (*v/v*) (HPLC grade solvents, Fisher Scientific Loughborough, UK), was added as an extractant solution. The mixture was shaken for exactly 1.5 min in a vortex agitator (Genious 3, IKA-Werke GmbH & Co, Staufen, Germany). Then, the tube was placed in the ultrasonic bath for 15 min at room temperature (RT) and centrifuged (Heraeus Multifuge 3S, Thermo Fisher Scientific, Waltham, MA, USA) at 4000 rpm for 25 min at RT. The supernatant was filtered with a 5 mL plastic syringe through a 0.45 μm PVDF filter and transferred to a new tube (falcon type). A glass syringe of 2.5 mL, (Gastlight, 1002TLL, Hamilton Company, Reno, NV, USA) was used to transfer 15 mL of the extract to a glass spherical flask and evaporate to dryness with a RotaVapor at 40 °C under vacuum (Büchi AG, Flawil, Switzerland). The solid extract was transferred to a 2 mL eppendorf (Eppendorf AG, Hamburg, Germany) and dried using a centrifugal evaporator with vacuum (Concentrator Plus, Eppendorf AG, Germany). Samples, after their extraction, were stored in a freezer at −20 °C until analysis.

### 3.3. NMR Analysis

#### 3.3.1. Preparation of NMR Samples

A stock solution of methanol-*d*_4_ (99.8% D, Euriso-Top GmbH, Saarbrücken, Germany) with 0.02% *v/v* HMDSO (NMR grade, ≥ 99.5%, Sigma-Aldrich Corporation, St. Louis, MO, USA) as an IS and a line-shape indicator was prepared. Each sample was dissolved within the eppendorf in 700 μL of the deuterated stock solution, sonicated for 30 s and centrifuged (MIKRO 200R, Andreas Hettich GmbH & Co. Tuttlingen, Germany) at 14,000 rpm for 5 min. The supernatant (550 μL) was transferred via a 1-mL Hamilton glass syringe and placed in a 5 mm NMR tube (D400-5-7) with a PTFE cap (both obtained by Deutero GmbH, Kastellaun, Germany).

#### 3.3.2. NMR Experimental Parameters

^1^H NMR experiments were recorded at 305 K on a Bruker AVANCE III 600 NMR spectrometer (Bruker GmbH, Rheinstetten, Germany) operating at the proton frequency of 600.13 MHz (B0 = 14.1 T) and equipped with a z-gradient inverse detection 5-mm probe and a BCU for temperature control. Spectra were recorded with the help of a 60-place sample changer (B-ACS-60), using the IconNMR automation software by Bruker. The following conditions were used for the acquisition: number of scans, 64; π/2 pulse, ~8 μs; time domain (TD), 64k data points; acquisition time, 2.73 s; relaxation delay, 3.17 s; spectral width, 12019.2 Hz; and mixing time, 0.060 s. The spectra were obtained by the Fourier transformation (FT) of the free induction decay (FID) by applying an exponential multiplication with a line-broadening factor (lb) of 0.3 Hz and zero-filling (size = 256K) procedure. Resulting spectra were manually phased and baseline-corrected using a polynomial function in the Bruker TopSpin software (version 4.0.6). Chemical shifts were reported with respect to the IS’s signal set at 0 ppm.

### 3.4. Computational Processing and Multivariate Analysis

NMR raw data were inserted in the MATLAB suite (version R2018b) for further processing. Initially, spectra were aligned using the icoshift tool (Version 3.0 beta) and a targeted selection of intervals optimized depending on the sample type. NMR spectra (spectral width from −0.5 to 11 ppm) were segmented into 1,111 bins with a bin size of 0.01 ppm for MVA and into 11,101 bins with a bin size of 0.001 ppm for the STOCSY experiments. Data were normalized using an *in-house* routine and the area of the IS’s peak was used as a reference value. Solvent signals (methanol-d_4_, methanol, water) and the IS signal, as well as the region from 8.5 to 9.6 ppm were removed for MVA. In particular, bins of the last segment mentioned were summed together as there were only minor peaks surfacing from the noise. As a result, 753 variables were obtained after data processing in MATLAB.

Data with the 0.01 ppm bin size were extracted and imported into SIMCA v. 14.1 (Umetrics, Umea, Sweden), where they were subjected to MVA and specifically the PCA, PLS-DA and OPLS-DA methods of analysis [85]. Prior to PCA, data were scaled using Pareto scaling along with logarithmic transformation and then mean-centered. In Pareto scaling, each variable is given a variance numerically equal to its initial standard deviation instead of unit variance, with the scaling weight being 1/$\sqrt{s_{k}}$, where s_k_ is the standard deviation of the k variable. With mean-centering, the average value of each variable is calculated and then subtracted from the data, hence improving the interpretability of the model.

Data with the 0.001 ppm bin size were used for the 1D STOCSY analyses with an *in-house* routine in the MATLAB suite. In theory, given that the spectrometer conditions are kept identical between samples, because the different resonance intensities from a single molecule will always have the same ratio, the relative intensities will be totally correlated. STOCSY utilizes this principle to generate a pseudo-NMR spectrum that displays the correlation among the intensities of various peaks across the entire sample [43]. Specifically, in this study, STOCSY calculates the correlation coefficient between a defined “driver” peak and all other signals in the spectrum. A threshold of 0.75 for the correlation coefficient was set on the whole dataset and it was kept at the same level for all generated pseudo-spectra. Therefore, our two-step approach uses OPLS-DA to extract NMR shift values that are then cross-combined with the STOCSY analysis, in order to strengthen the identification of the molecules responsible for any metabolic alteration.

Correlation analysis and heat maps were performed for data visualization using MetaboAnalyst 4.0. A “warm-to-cool” color spectrum was used in order to reveal the metabolic alterations of the selected metabolites in the samples set in a column hierarchical cluster structure of the data matrix [86].

## 4. Conclusions

Edible olives, a valuable element of the Mediterranean diet, keep rising as a nutritional choice worldwide, while the level of fraud has always been an issue in the food industry. From geographical origin to variety and processing method, quality should be a prerequisite for the last border, the consumer. The protocols issued by the IOC so far only cover the important, yet inadequate, sensory aspect. In the current study, NMR-based metabolomics was combined with MVA for the first time in the quality assessment of edible olives. Several markers were successfully identified for the discrimination between samples with a different geographical origin, variety and processing method. Furthermore, the—novel in natural products—tool of STOCSY was applied, revealing certain peak correlations, which in collaboration with the literature led to an unprecedented peak assignment for these compounds in the total extract of such samples. This work is an important contribution to the confrontation of food mislabeling, intentional or accidental. More samples from different varieties, regions and processing methods should be analyzed to expand the versatility of the models created and more unknown compounds have to be identified in the future.

## Figures and Tables

**Table 1:** List of analyzed samples of edible olives along with their metadata (classification).

	Region	Subregion	Variety	Processing Type	Sample Number
1	Makedonia	Chalkidiki	Chalkidikis	Spanish (green)	9
2		Kavala	Chalkidikis		6
3	Sterea Ellada	Magnesia	Konservolia	Greek (black)	6
4		Fthiotida	Konservolia		9
5		Fthiotida	Kalamon		1
6		Aitoloakarnania	Kalamon		14
7	Peloponnese	Messinia	Kalamon		10
8		Lakonia	Kalamon		5

**Table 2:** ^1^H NMR chemical shifts and assignments for edible olives’ metabolites identified. Respective reference per compound is also presented.

No	Compound	δ^1^H (Multiplicity, *J* in Hz, Assignment)	Ref.
**1**	**Tyr**	6.96 (d, *J* = 8.5 Hz, H-2, H-6), 6.64 (d, *J* = 8.5 Hz, H-3, H-5), 3.62 (t, *J* = 7.2 Hz, H-8), 2.65 (t, *J* = 7.2 Hz, H-7)	[56]
**2**	**HT**	6.62 (d, *J* = 8.0 Hz, H-5), 6.59 (d, *J* = 1.9 Hz, H-2), 6.46 (dd, *J* = 1.9/8.0 Hz, H-6), 3.61 (t, *J* = 7.2 Hz, H-8), 2.60 (t, *J* = 7.2 Hz, H-7)	[57]
**3**	**Ver**	7.53 (d, *J* = 15.9 Hz, H-7′’’), 7.00 (d, *J* = 2.0 Hz, H-2′’’), 6.89 (dd, *J* = 2.0/8.2 Hz, H6′’’), 6.72 (d, *J* = 8.2 Hz, H-5′’’), 6.63 (m, H-2), 6.61 (m, H-5), 6.50 (dd, *J* = 2.0/8.0 Hz, H-6), 6.21 (d, *J* = 15.9 Hz, H-8′’’), 5.13 (d, *J* = 1.7 Hz, H-1′’), 4.85 (m, H-4′), 4.32 (d, *J* = 8.0 Hz, H-1′), 3.98-2.73(m, H-7, H-8, H-2′, H-3′, H-5′, H-6′, H-2′’, H-3′’, H-4′’, H-5′’), 1.03 (d, *J* = 6.2 Hz, H-6′’)	[58]
**4**	**Lut**	7.32 (dd, *J* = 2.2/9.0 Hz, H-6′), 7.32 (d, *J* = 2.2 Hz, H-2′), 6.85 (d, *J* = 9.0 Hz, H-5′), 6.48 (s, H-3), 6.39 (d, *J* = 2.1 Hz, H-8), 6.15 (d, *J* = 2.1 Hz, H-6)	[59]
**5**	**Quer**	7.67 (d, *J* = 2.2 Hz, H-2′), 7.57 (dd, *J* = 2.2/8.5 Hz, H-6′), 6.83 (d, *J* = 8.5 Hz, H-5′), 6.34 (d, *J* = 2.0 Hz, H-8), 6.13 (d, *J* = 2.0 Hz, H-6)	[60]
**6**	**MA**	5.19 (brt, *J* = 3.6 Hz, H-12), 3.55 (m, H-2), 2.85 (d, *J* = 9.6 Hz, H-3), 2.80 (dd, *J* = 4.1/14.1 Hz, H-18), 1.10 (s, H-27), 0.95 (s, H-24), 0.94 (s, H-23), 0.88 (s, H-30), 0.85 (s, H-29), 0.76 (s, H-26), 0.75 (s, H-25)	[61]
**7**	**OA**	5.18 (brt, *J* = 3.5 Hz, H-12), 3.08 (dd, *J* = 4.9/11.7 Hz, H-3), 2.79 (dd, *J* = 4.1/14.3 Hz, H-18), 1.09 (s, H-27), 0,91 (s, H-23), 0.88 (s, H-25, H-30), 0.85 (s, H-29), 0.76 (s, H-26), 0.71 (s, H-24)	[61]
**8**	**SA**	2.46 (s, H-2, H-3)	[62]
**9**	**LA**	4.04 (q, *J* = 6.9 Hz, H-2), 1.29 (d, *J* = 6.9 Hz, H-3)	[62]
**10**	**PA**	2.13 (q, *J* = 7.6 Hz, H-2), 1.03 (t, *J* = 7.6 Hz, H-3)	[63]
**11**	**AA**	1.85 (s, H-2)	[64]
**12**	**FA**	8.45 (s)	[64]
**13**	**TAGs**	5.28 (m, olefinic protons), 5,25 (m, glyceryl group, H-2), 4.08 (dd, *J* = 4.4/11.4 Hz, glyceryl group, H-1a, H-3a), 4.01 (dd, 6.2/11.4 Hz, glyceryl group, H-1b, H-3b), 2.20 (t, *J =* 7.5 Hz, acyl groups, -OCO-CH_2_-), 2.02-1.95 (m, acyl groups, allylic protons), 1.54 (m, acyl groups, -OCO-CH_2-_CH_2__-_), 1.34-1.18 (m, acyl groups, -(CH_2_)_n_-), 0.83 (t, 7.0, saturated/ monosaturated methyl group)	[65]
**14**	Lin*	5.28 (m, olefinic protons), 5,25 (m, glyceryl group, H-2), 4.08 (dd, J = 4.4/11.4 Hz, glyceryl group, H-1a, H-3a), 4.01 (dd, 6.2/11.4 Hz, glyceryl group, H-1b, H-3b), 2.71 (t, *J =* 6.7 Hz, H-11, bis-allylic proton), 2.20 (t, *J* = 7.5 Hz, acyl groups, -OCO-CH_2_-), 2.02-1.95 (m, acyl groups, allylic protons), 1.54 (m, acyl groups, -OCO-CH_2_-CH_2_-), 1.34-1.18 (m, acyl groups, -(CH_2_)_n_-), 0.84 (t, *J =* 7.0 Hz, H-18)	[65]
**15**	G**	3.59 (m, H-2), 3.53 (dd, *J* = 4.8/11.2 Hz, H-1a, H-3a), 3.46 (dd, *J* = 6.0/11.1 Hz, H-1b, H-3b)	[66]

^1^ brt, broad triplet; d, doublet; dd, doublet of doublets; m, multiplet; q, quartet; s, singlet; t, triplet.

* Indicative signals of bound linoleic acid; ** indicative signals of free glycerol.

## Supplementary Materials

The following are available online. Figure S1. PLS-DA scores plot of the geographical origin parameter with the respective conducted permutation tests; Figure S2. OPLS-DA scores plots of the geographical origin parameter with their respective S-plots; Figure S3. PLS-DA scores plot of the variety parameter with the respective conducted permutation tests; Figure S4. OPLS-DA scores plots of the geographical origin parameter with their respective S-plots; Figure S5. PLS-DA scores plot of the processing parameter with the respective conducted permutation tests; Figure S6. OPLS-DA scores plot of the processing parameter with its respective S-plot; Figure S7. Box plots of the remaining markers in the origin parameter; Figure S8. Box plots of the remaining markers in the variety parameter; Table S1. Description of samples’ characteristics; Table S2. VIPs lists from all OPLS-DA models; Table S3. T-test applied at the processing method parameter, Greek vs. Spanish.

## References

[1] International Olive Council Olives De Table - Table Olives - Consumption. https://www.internationaloliveoil.org/wp-content/uploads/2020/01/OT-W901-29-11-2019-C.pdf.

[2] International Olive Council Key figures on the world market for table olives. https://www.internationaloliveoil.org/wp-content/uploads/2019/12/NEWSLETTER_144_ENGLISH.pdf.

[3] Ramírez E., Brenes M., De Castro A., Romero C., Medina E. (2017). Oleuropein hydrolysis by lactic acid bacteria in natural green olives. LWT. - Food Sci. Technol..

[4] Johnson R.L., Mitchell A.E. (2018). Reducing phenolics related to bitterness in table olives. J. Food Qual..

[5] Kailis S.G. (2016). Olives. Encycl. Appl. Plant Sci..

[6] Ryan D., Robards K. (1998). Phenolic compounds in olives. Analyst.

[7] Serreli G., Incani A., Atzeri A., Angioni A., Campus M., Cauli E., Zurru R., Deiana M. (2017). Antioxidant effect of natural table olives phenolic extract against oxidative stress and membrane damage in enterocyte-like cells. J. Food Sci..

[8] Gambino C.M., Accardi G., Aiello A., Candore G., Dara-Guccione G., Mirisola M., Procopio A., Taormina G., Caruso C. (2017). Effect of extra virgin olive oil and table olives on the immuneinflammatory responses: Potential clinical applications. Endocr. Metab. Immune Disord. - Drug Targets.

[9] Accardi G., Aiello A., Gargano V., Gambino C.M., Caracappa S., Marineo S., Vesco G., Carru C., Zinellu A., Zarcone M. (2016). Nutraceutical effects of table green olives: A pilot study with Nocellara del Belice olives. Immun. Ageing.

[10] Lozano-Mena G., Sánchez-González M., Juan M.E., Planas J.M. (2014). Maslinic acid, a natural phytoalexin-type triterpene from olives - A promising nutraceutical?. Molecules.

[11] Jiang L.Q., Takamura H. (2013). Radical-scavenging compounds in olive fruits and their changes during table olive preparation. Appl. Mech. Mater..

[12] Emilia Juan M., Wenzel U., Daniel H., Planas J.M. (2010). Olive Fruit Extracts and HT-29 Human Colon Cancer Cells.

[13] Boskou D., Boskou D. (2015). Olive fruit, table olives, and olive oil bioactive constituents. Olive and Olive Oil Bioactive Constituents.

[14] Fiehn O. (2002). Metabolomics - The link between genotypes and phenotypes. Plant Mol. Biol..

[15] Durante M., Tufariello M., Tommasi L., Lenucci M.S., Bleve G., Mita G. (2018). Evaluation of bioactive compounds in black table olives fermented with selected microbial starters. J. Sci. Food Agric..

[16] Boskou D., Blekas G., Tsimidou M. (2005). Phenolic compounds in olive oil and olives. Curr. Top. Nutraceutical Res..

[17] International Olive Council Method: Sensory Analysis of Table Olives. https://www.internationaloliveoil.org/wp-content/uploads/2019/11/COI-OT-MO.-1-Rev.2-2011-Eng.pdf.

[18] Blekas G., Vassilakis C., Harizanis C., Tsimidou M., Boskou D.G. (2002). Biophenols in table olives. J. Agric. Food Chem..

[19] López-López A., Montaño A., Garrido-Fernández A. (2010). Nutrient Profiles of Commercial Table Olives: Fatty Acids, Sterols, and Fatty Alcohols.

[20] Sahan Y., Cansev A., Gulen H. (2013). Effect of processing techniques on antioxidative enzyme activities, antioxidant capacity, phenolic compounds, and fatty acids of table olives. Food Sci. Biotechnol..

[21] (2014). European Union Commission Implementing Regulation (EU) No 668/2014. Off. J. Eur. Union.

[22] Luykx D.M.A.M., Van Ruth S.M. (2008). An overview of analytical methods for determining the geographical origin of food products. Food Chem..

[23] Böhme K., Calo-Mata P., Barros-Velázquez J., Ortea I. (2019). Recent applications of omics-based technologies to main topics in food authentication. TrAC Trends Anal. Chem..

[24] Versari A., Laurie V.F., Ricci A., Laghi L., Parpinello G.P. (2014). Progress in authentication, typification and traceability of grapes and wines by chemometric approaches. Food Res. Int..

[25] Steinmann D., Ganzera M. (2011). Recent advances on HPLC/MS in medicinal plant analysis. J. Pharm. Biomed. Anal..

[26] Soler C., Picó Y. (2007). Recent trends in liquid chromatography-tandem mass spectrometry to determine pesticides and their metabolites in food. TrAC Trends Anal. Chem..

[27] De Castro A., Sánchez A.H., López-López A., Cortés-Delgado A., Medina E., Montaño A. (2018). Microbiota and metabolite profiling of spoiled spanish-style green table olives. Metabolites.

[28] López A., Montaño A., García P., Garrido A. (2006). Fatty acid profile of table olives and its multivariate characterization using unsupervised (PCA) and supervised (DA) chemometrics. J. Agric. Food Chem..

[29] López-López A., Jiménez-Araujo A., García-García P., Garrido-Fernández A. (2007). Multivariate analysis for the evaluation of fiber, sugars, and organic acids in commercial presentations of table olives. J. Agric. Food Chem..

[30] López A., García P., Garrido A. (2008). Multivariate characterization of table olives according to their mineral nutrient composition. Food Chem..

[31] Vallone M., Alleri M., Bono F., Catania P. (2019). Use of a portable VIS NIR device to predict table olives quality. Chem. Eng. Trans..

[32] Boskou G. (2010). Antioxidant Capacity and Phenolic Profile of Table Olives from the Greek Market.

[33] Gougoulias N., Giurgiulescu L., Vagelas I., Wogiatzi E., Ntalla M.N. (2017). Changes in total phenol content and antioxidant activity of greek table olive cultivar amfissis during maturation. Stud. Univ. Babes-Bolyai Chem..

[34] Mitsopoulos G., Papageorgiou V., Komaitis M., Hagidimitriou M. (2016). Total phenolic content, phenolic profile and antioxidant activity of leaves and drupes in major Greek olive varieties. Not. Bot. Horti. Agrobot..

[35] Mastralexi A., Mantzouridou F.T., Tsimidou M.Z. (2019). Evolution of safety and other quality parameters of the greek pdo table olives “prasines elies chalkidikis” during industrial scale processing and storage. Eur. J. Lipid Sci. Technol..

[36] Panagou E.Z., Tassou C.C., Skandamis P.N. (2006). Physicochemical, microbiological, and organoleptic profiles of Greek table olives from retail outlets. J. Food Prot..

[37] Alexandraki V., Georgalaki M., Papadimitriou K., Anastasiou R., Zoumpopoulou G., Chatzipavlidis I., Papadelli M., Vallis N., Moschochoritis K., Tsakalidou E. (2014). Determination of triterpenic acids in natural and alkaline-treated Greek table olives throughout the fermentation process. LWT—Food Sci. Technol..

[38] Zoidou E., Melliou E., Gikas E., Tsarbopoulos A., Magiatis P., Skaltsounis A.L. (2010). Identification of throuba thassos, a traditional Greek table olive variety, as a nutritional rich source of oleuropein. J. Agric. Food Chem..

[39] Rotondo A., Mannina L., Salvo A. (2019). Multiple assignment recovered analysis (MARA) NMR for a direct food labeling: The case study of olive oils. Food Anal. Methods.

[40] Spiteri M., Jamin E., Thomas F., Rebours A., Lees M., Rogers K.M., Rutledge D.N. (2015). Fast and global authenticity screening of honey using 1H-NMR profiling. Food Chem..

[41] Jakes W., Gerdova A., Defernez M., Watson A.D., McCallum C., Limer E., Colquhoun I.J., Williamson D.C., Kemsley E.K. (2015). Authentication of beef versus horse meat using 60 MHz 1H NMR spectroscopy. Food Chem..

[42] Halabalaki M., Vougogiannopoulou K., Mikros E., Skaltsounis A.L. (2014). Recent advances and new strategies in the NMR-based identification of natural products. Curr. Opin. Biotechnol..

[43] Cloarec O., Dumas M.-E., Craig A., Barton R.H., Trygg J., Hudson J., Blancher C., Gauguier D., Lindon J.C., Holmes E. (2005). Statistical total correlation spectroscopy: An exploratory approach for latent biomarker identification from metabolic 1 H NMR data sets. Anal. Chem..

[44] Boka V.I., Stathopoulou K., Benaki D., Gikas E., Aligiannis N., Mikros E., Skaltsounis A.L. (2017). Could multivariate statistics exploit HPTLC and NMR data to reveal bioactive compounds? The case of *Paeonia mascula*. Phytochem. Lett..

[45] Yilmaz A., Nyberg N.T., Jaroszewski J.W. (2012). Extraction of alkaloids for NMR-based profiling: Exploratory analysis of an archaic Cinchona bark collection. Planta Med..

[46] Guldbrandsen N., Kostidis S., Schäfer H., De Mieri M., Spraul M., Skaltsounis A.L., Mikros E., Hamburger M. (2015). NMR-based metabolomic study on isatis tinctoria: Comparison of different accessions, harvesting dates, and the effect of repeated harvesting. J. Nat. Prod..

[47] Freire R.T., Bero J., Beaufay C., Selegato D.M., Coqueiro A., Choi Y.H., Quetin-Leclercq J. (2019). Identification of antiplasmodial triterpenes from Keetia species using NMR-based metabolic profiling. Metabolomics.

[48] Kang J., Choi M.Y., Kang S., Hyuk N.K., Wen H., Chang H.L., Park M., Wiklund S., Hyo J.K., Sung W.K. (2008). Application of a ^1^H nuclear magnetic resonance (NMR) metabolomics approach combined with orthogonal projections to latent structure-discriminant analysis as an efficient tool for discriminating between Korean and Chinese herbal medicines. J. Agric. Food Chem..

[49] Lopez J.M., Cabrera R., Maruenda H. (2019). Ultra-clean pure shift ^1^H-NMR applied to metabolomics profiling. Sci. Rep..

[50] Bo Y., Feng J., Xu J.J., Huang Y., Cai H., Cui X., Dong J., Ding S., Chen Z. (2019). High-resolution pure shift NMR spectroscopy offers better metabolite discrimination in food quality analysis. Food Res. Int..

[51] Kew W., Goodall I., Uhrín D. (2019). Analysis of scotch whisky by ^1^H NMR and chemometrics yields insight into its complex chemistry. Food Chem..

[52] Ghanbari R., Anwar F., Alkharfy K.M., Gilani A.H., Saari N. (2012). Valuable nutrients and functional bioactives in different parts of olive (*Olea europaea* L.)-A review. Int. J. Mol. Sci..

[53] Evangelou E., Kiritsakis K., Sakellaropoulos N., Kiritsakis A. (2018). Table olives production, postharvest processing, and nutritional qualities. Handbook of Vegetables and Vegetable Processing.

[54] Rotondo A., Salvo A., Gallo V., Rastrelli L., Dugo G. (2017). Quick unreferenced NMR quantification of Squalene in vegetable oils. Eur. J. Lipid Sci. Technol..

[55] Danezis G.P., Tsagkaris A.S., Camin F., Brusic V., Georgiou C.A. (2016). Food authentication: Techniques, trends & emerging approaches. TrAC Trends Anal. Chem..

[56] Sugiyama Y., Ito Y., Suzuki M., Hirota A. (2009). Indole derivatives from a marine sponge-derived yeast as DPPH radical scavengers. J. Nat. Prod..

[57] Kalampaliki A.D., Giannouli V., Skaltsounis A.L., Kostakis I.K. (2019). A three-step, gram-scale synthesis of hydroxytyrosol, hydroxytyrosol acetate, and 3,4-dihydroxyphenylglycol. Molecules.

[58] Wang Y., Zhou S., Xu G., Gao Y. (2015). Interference of phenylethanoid glycosides from cistanche tubulosa with the MTT assay. Molecules.

[59] Wang L., Li X., Zhang S., Lu W., Liao S., Liu X., Shan L., Shen X., Jiang H., Zhang W. (2012). Natural products as a gold mine for selective matrix metalloproteinases inhibitors. Bioorg. Med. Chem..

[60] Tasnuva S.T., Qamar U.A., Ghafoor K., Sahena F., Jahurul M.H.A., Rukshana H., Juliana M.J., Al-juhaimi F.Y., Jalifah L., Jalal K.C.A. (2019). α -glucosidase inhibitors isolated from *Mimosa pudica* L.. Nat. Prod. Res..

[61] Lima R.D.C.L., Kongstad K.T., Kato L., Das Silva M.J., Franzyk H., Staerk D. (2018). High-resolution PTP1B inhibition profiling combined with HPLC-HRMS-SPE-NMR for identification of PTP1B inhibitors from Miconia albicans. Molecules.

[62] Albergamo A., Rotondo A., Salvo A., Pellizzeri V., Bua D.G., Maggio A., Cicero N., Dugo G., Albergamo A., Rotondo A. (2017). Metabolite and mineral profiling of “ Violetto di Niscemi ” and “ Spinoso di Menfi ” globe artichokes by H-NMR and ICP-MS. Nat. Prod. Res..

[63] Monguchi Y., Ichikawa T., Nozaki K., Kihara K., Yamada Y., Miyake Y., Sawama Y., Sajiki H. (2015). Development of chelate resin-supported palladium catalysts for chemoselective hydrogenation. Tetrahedron.

[64] Gottlieb H.E., Kotlyar V., Nudelman A. (1997). NMR chemical shifts of common laboratory solvents as trace impurities. J. Org. Chem..

[65] Ruiz-Aracama A., Goicoechea E., Guillén M.D. (2017). Direct study of minor extra-virgin olive oil components without any sample modification. 1H NMR multisupression experiment: A powerful tool. Food Chem..

[66] Rathnayake G.R.N., Kumar N.S., Jayasinghe L., Araya H., Fujimoto Y. (2018). Chemical investigation of metabolites produced by an endophytic fungi Phialemonium curvatum from the leaves of Passiflora edulis. Nat. Prod. Res..

[67] Dugo G., Rotondo A., Mallamace D., Cicero N., Salvo A., Rotondo E., Corsaro C. (2015). Enhanced detection of aldehydes in extra-virgin olive oil by means of band selective NMR spectroscopy. Phys. A Stat. Mech. Its Appl..

[68] Klikarová J., Rotondo A., Cacciola F., Česlová L., Dugo P., Mondello L., Rigano F. (2019). The phenolic fraction of italian extra virgin olive oils: Elucidation through combined liquid chromatography and NMR approaches. Food Anal. Methods.

[69] Moreno-González R., Juan M.E., Planas J.M. (2019). Table olive polyphenols: A simultaneous determination by liquid chromatography–mass spectrometry. J. Chromatogr. A.

[70] Kanakis P., Termentzi A., Michel T., Gikas E., Halabalaki M., Skaltsounis A.L. (2013). From olive drupes to olive oil. An HPLC-orbitrap-based qualitative and quantitative exploration of olive key metabolites. Planta Med..

[71] Cabrera-Bañegil M., Schaide T., Manzano R., Delgado-Adámez J., Durán-Merás I., Martín-Vertedor D. (2017). Optimization and validation of a rapid liquid chromatography method for determination of the main polyphenolic compounds in table olives and in olive paste. Food Chem..

[72] Ayeleso T.B. (2017). Oleanolic acid and its derivatives: Biological activities and therapeutic potential in chronic diseases. Molecules.

[73] Pasqualone A., Nasti R., Montemurro C., Gomes T. (2014). Effect of natural-style processing on the oxidative and hydrolytic degradation of the lipid fraction of table olives. Food Control.

[74] Alves E., Melo T., Barros M.P., Ros M.R.M., Domingues P. (2019). Lipidomic Profiling of the Olive (*Olea europaea L*.) fruit towards its valorisation as a functional food: In-depth identification of triacylglycerols and polar. Molecules.

[75] Tsantili E. (2014). Quality attributes and their relations in fresh black ripe “Kalamon” olives (*Olea europaea L*.) for table use - phenolic compounds and total antioxidant capacity. Int. J. Food Sci. Technol..

[76] Gentile L., Uccella N.A. (2014). Selected bioactives from callus cultures of olives (*Olea europaea L.* Var. Coratina) by LC-MS. Food Res. Int..

[77] Bastoni L., Bianco A., Piccioni F., Uccella N. (2001). Biophenolic profile in olives by nuclear magnetic resonance. Food Chem..

[78] Bouaziz M., Jemai H., Khabou W., Sayadi S. (2010). Oil content, phenolic profiling and antioxidant potential of Tunisian olive drupes. J. Sci. Food Agric..

[79] Brenes M., Medina E., García A., Romero C., De Castro A. (2010). Olives and Olive Oil Compounds Active Against Pathogenic Microorganisms.

[80] Emília Juan M., Wenzel U., Daniel H., Planas J.M. (2010). Cancer Chemopreventive Activity of Hydroxytyrosol: A Natural Antioxidant from Olives and Olive Oil.

[81] (2011). Scientific Opinion on the substantiation of health claims related to polyphenols in olive and protection of LDL particles from oxidative damage (ID **1333**, *1638*, 1639, 1696, 2865), maintenance of normal blood HDL-cholesterol concentrations (ID 1639), mainte. Efsa J..

[82] Montano A., Sanchez A.H., Casado F.J., De Castro A., Rejano L. (2003). Chemical profile of industrially fermented green olives of different varieties. Food Chem..

[83] Sabatini N., Marsilio V. (2008). Volatile compounds in table olives (*Olea Europaea L*., Nocellara del Belice cultivar). Food Chem..

[84] International Olive Council (2017). Determination of Biophenols in Oive Oils by HPLC. https://www.internationaloliveoil.org/wp-content/uploads/2019/11/COI-T.20-Doc.-No-29-Rev-1-2017.pdf.

[85] Eriksson L., Byrne T., Johansson E., Trygg J., Wikström C. (2013). Multi- and Megavariate Data Analysis Basic Principles and Applications.

[86] Xia J., Wishart D.S. (2016). Using metaboanalyst 3.0 for comprehensive metabolomics data analysis. Curr. Protoc. Bioinforma..

