# Supplementary material for: NMR-Based Metabolic Profiling of Edible Olives—Determination of Quality Parameters
Source: Molecules. 2020 Jul 23;25(15):3339. doi: 10.3390/molecules25153339 (PMC7436060; doi:10.3390/molecules25153339)
Supplement: Supplementary file 1 [file molecules-25-03339-s001.docx]

*Supplementary Materials*

NMR-based metabolic profiling of edible olives – Determination of quality parameters

Stavros Beteinakis ^1^, Anastasia Papachristodoulou ^1^, Georgia Gogou ^1,2^, Sotirios Katsikis ^1^, Emmanuel Mikros ^3^, Maria Halabalaki ^1,^ *

^1^ Division of Pharmacognosy and Natural Products Chemistry, Department of Pharmacy, National and Kapodistrian University of Athens, Panepistimiopolis, Zografou, 15771, Athens, Greece;

E-Mails: sbeteinakis@pharm.uoa.gr (S.B.); anpapac@pharm.uoa.gr (A.P.); sotirisk@gmail.com (S.K.); mariahal@pharm.uoa.gr (M.H.)

^2^ Laboratory of Cellular Immunology, Department of Microbiology, Hellenic Pasteur Institute, 127 Vas. Sofias av., 11521 Athens, Greece; E-mail: gogo94go122@hotmail.com

^3^ Division of Pharmaceutical Chemistry, Department of Pharmacy, National and Kapodistrian University of Athens, Panepistimiopolis Zografou, 15771, Athens, Greece; E-mail: mikros@pharm.uoa.gr

* Correspondence: mariahal@pharm.uoa.gr; Tel.: +30-210-7274781 (M.H)

Content

**Table S1.** Description of samples’ chracteristics. 2

**Figure S1.** PLS-DA scores plot of the geographical origin parameter with the respective conducted permutation tests. 3

**Figure S2.** OPLS-DA scores plots of the geographical origin parameter with their respective S-plots. 4

**Figure S3.** PLS-DA scores plot of the variety parameter with the respective conducted permutation tests. 5

**Figure S4.** OPLS-DA scores plots of the geographical origin parameter with their respective S-plots. 6

**Figure S5.** PLS-DA scores plot of the processing parameter with the respective conducted permutation tests. 7

**Figure S6.** OPLS-DA scores plot of the processing parameter with its respective S-plot. 7

**Table S2.** VIPs lists from all OPLS-DA models. 8

**Figure S7.** Box-plots of the remaining markers in the origin parameter. 15

**Figure S8.** Box-plots of the remaining markers in the variety parameter. 16

**Table S3.** T-test applied at the processing method parameter, Greek vs Spanish. 16

**Table S1.** Samples’ metadata

| **Observation ID** | **Region** | **Subregion** | **Variety** | **Colour** | **Fermentation** |
| --- | --- | --- | --- | --- | --- |
| GMKa_1 | Peloponnese | Messinia | Kalamon | Black | Greek |
| GLKa_2 | Peloponnese | Lakonia | Kalamon | Black | Greek |
| GMKa_3 | Peloponnese | Messinia | Kalamon | Black | Greek |
| GMKa_4 | Peloponnese | Messinia | Kalamon | Black | Greek |
| GLKa_5 | Peloponnese | Lakonia | Kalamon | Black | Greek |
| GMKa_6 | Peloponnese | Messinia | Kalamon | Black | Greek |
| GLKa_7 | Peloponnese | Lakonia | Kalamon | Black | Greek |
| GFKa_8 | Sterea Ellada | Fthiotida | Kalamon | Black | Greek |
| GAKa_9 | Sterea Ellada | Aitol/nia | Kalamon | Black | Greek |
| GAKa_10 | Sterea Ellada | Aitol/nia | Kalamon | Black | Greek |
| GMKa_11 | Peloponnese | Messinia | Kalamon | Black | Greek |
| GMKa_12 | Peloponnese | Messinia | Kalamon | Black | Greek |
| GMKa_13 | Peloponnese | Messinia | Kalamon | Black | Greek |
| GMKa_14 | Peloponnese | Messinia | Kalamon | Black | Greek |
| GMKa_15 | Peloponnese | Messinia | Kalamon | Black | Greek |
| GAKa_16 | Sterea Ellada | Aitol/nia | Kalamon | Black | Greek |
| GAKa_17 | Sterea Ellada | Aitol/nia | Kalamon | Black | Greek |
| GAKa_18 | Sterea Ellada | Aitol/nia | Kalamon | Black | Greek |
| GAKa_19 | Sterea Ellada | Aitol/nia | Kalamon | Black | Greek |
| GAKa_20 | Sterea Ellada | Aitol/nia | Kalamon | Black | Greek |
| GAKa_21 | Sterea Ellada | Aitol/nia | Kalamon | Black | Greek |
| GAKa_22 | Sterea Ellada | Aitol/nia | Kalamon | Black | Greek |
| GAKa_23 | Sterea Ellada | Aitol/nia | Kalamon | Black | Greek |
| GAKa_24 | Sterea Ellada | Aitol/nia | Kalamon | Black | Greek |
| GAKa_25 | Sterea Ellada | Aitol/nia | Kalamon | Black | Greek |
| GAKa_26 | Sterea Ellada | Aitol/nia | Kalamon | Black | Greek |
| GAKa_27 | Sterea Ellada | Aitol/nia | Kalamon | Black | Greek |
| GLKa_28 | Peloponnese | Lakonia | Kalamon | Black | Greek |
| GLKa_29 | Peloponnese | Lakonia | Kalamon | Black | Greek |
| GMKa_16a | Peloponnese | Messinia | Kalamon | Black | Greek |
| GFK_1 | Sterea Ellada | Fthiotida | Konservolia | Black | Greek |
| GFK_2 | Sterea Ellada | Fthiotida | Konservolia | Black | Greek |
| GFK_3 | Sterea Ellada | Fthiotida | Konservolia | Black | Greek |
| GFK_4 | Sterea Ellada | Fthiotida | Konservolia | Black | Greek |
| GFK_5 | Sterea Ellada | Fthiotida | Konservolia | Black | Greek |
| GFK_6 | Sterea Ellada | Fthiotida | Konservolia | Black | Greek |
| GMK_1 | Sterea Ellada | Magnesia | Konservolia | Black | Greek |
| GMK_2 | Sterea Ellada | Magnesia | Konservolia | Black | Greek |
| GMK_3 | Sterea Ellada | Magnesia | Konservolia | Black | Greek |
| GMK_4 | Sterea Ellada | Magnesia | Konservolia | Black | Greek |
| GMK_5 | Sterea Ellada | Magnesia | Konservolia | Black | Greek |
| GMK_6 | Sterea Ellada | Magnesia | Konservolia | Black | Greek |
| GFK_7 | Sterea Ellada | Fthiotida | Konservolia | Black | Greek |
| GFK_8 | Sterea Ellada | Fthiotida | Konservolia | Black | Greek |
| GFK_9 | Sterea Ellada | Fthiotida | Konservolia | Black | Greek |
| SKX_1 | Makedonia | Kavala | Chalkidikis | Green | Spanish |
| SKX_2 | Makedonia | Kavala | Chalkidikis | Green | Spanish |
| SKX_3 | Makedonia | Kavala | Chalkidikis | Green | Spanish |
| SKX_4 | Makedonia | Kavala | Chalkidikis | Green | Spanish |
| SKX_5 | Makedonia | Kavala | Chalkidikis | Green | Spanish |
| SKX_6 | Makedonia | Kavala | Chalkidikis | Green | Spanish |
| SXX_1 | Makedonia | Chalkidiki | Chalkidikis | Green | Spanish |
| SXX_2 | Makedonia | Chalkidiki | Chalkidikis | Green | Spanish |
| SXX_3 | Makedonia | Chalkidiki | Chalkidikis | Green | Spanish |
| SXX_4 | Makedonia | Chalkidiki | Chalkidikis | Green | Spanish |
| SXX_5 | Makedonia | Chalkidiki | Chalkidikis | Green | Spanish |
| SXX_6 | Makedonia | Chalkidiki | Chalkidikis | Green | Spanish |
| SXX_7 | Makedonia | Chalkidiki | Chalkidikis | Green | Spanish |
| SXX_8 | Makedonia | Chalkidiki | Chalkidikis | Green | Spanish |
| SXX_9 | Makedonia | Chalkidiki | Chalkidikis | Green | Spanish |


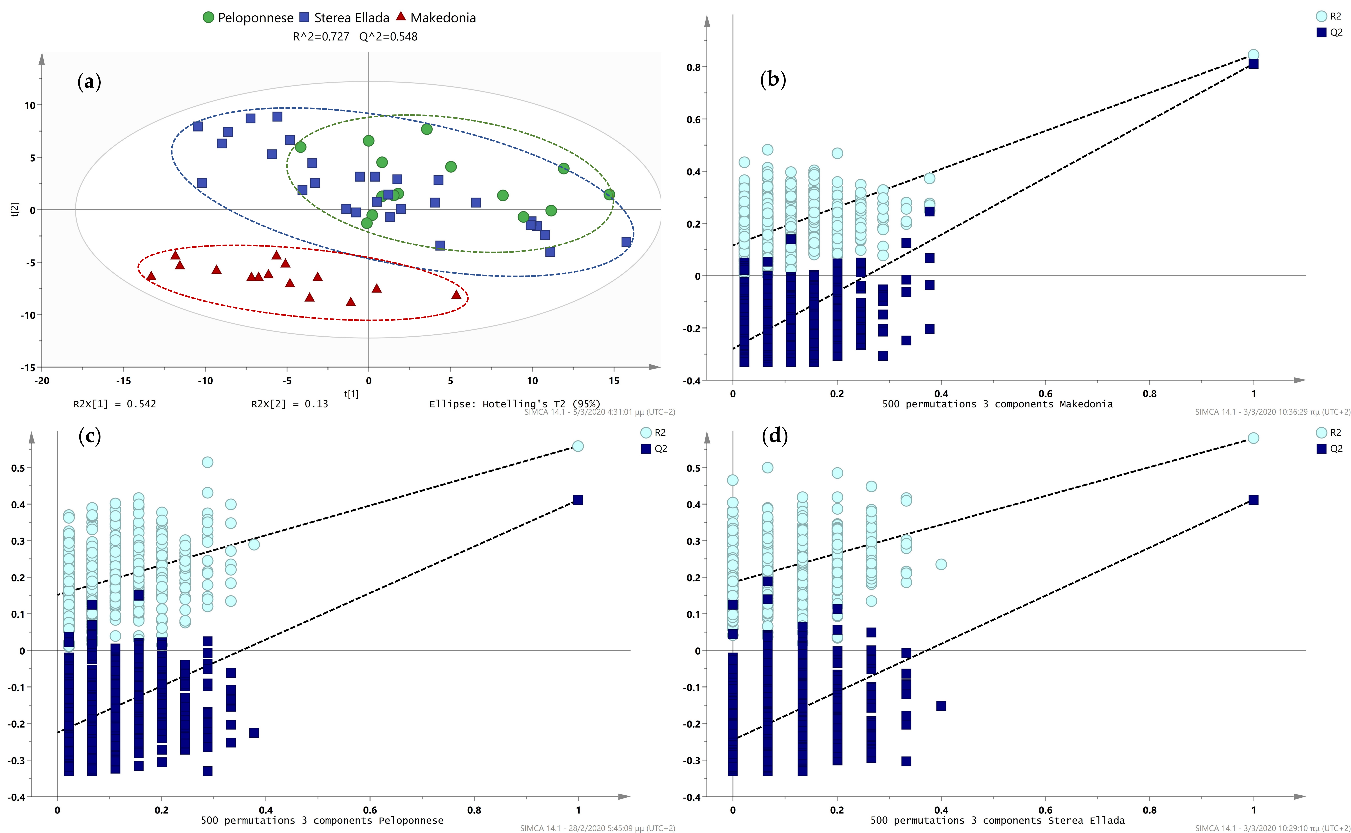


**Figure S1.** (**a**) PLS-DA scores plot with a clear distinction of samples from Makedonia; (**b**) Permutation test with 500 permutations performed at the presented PLS-DA model with samples from Makedonia, verifying the validity of the model.; (**c**) Respective permutation test with samples from Peloponnese; (**d**) Respective permutation test with samples from Sterea Ellada.


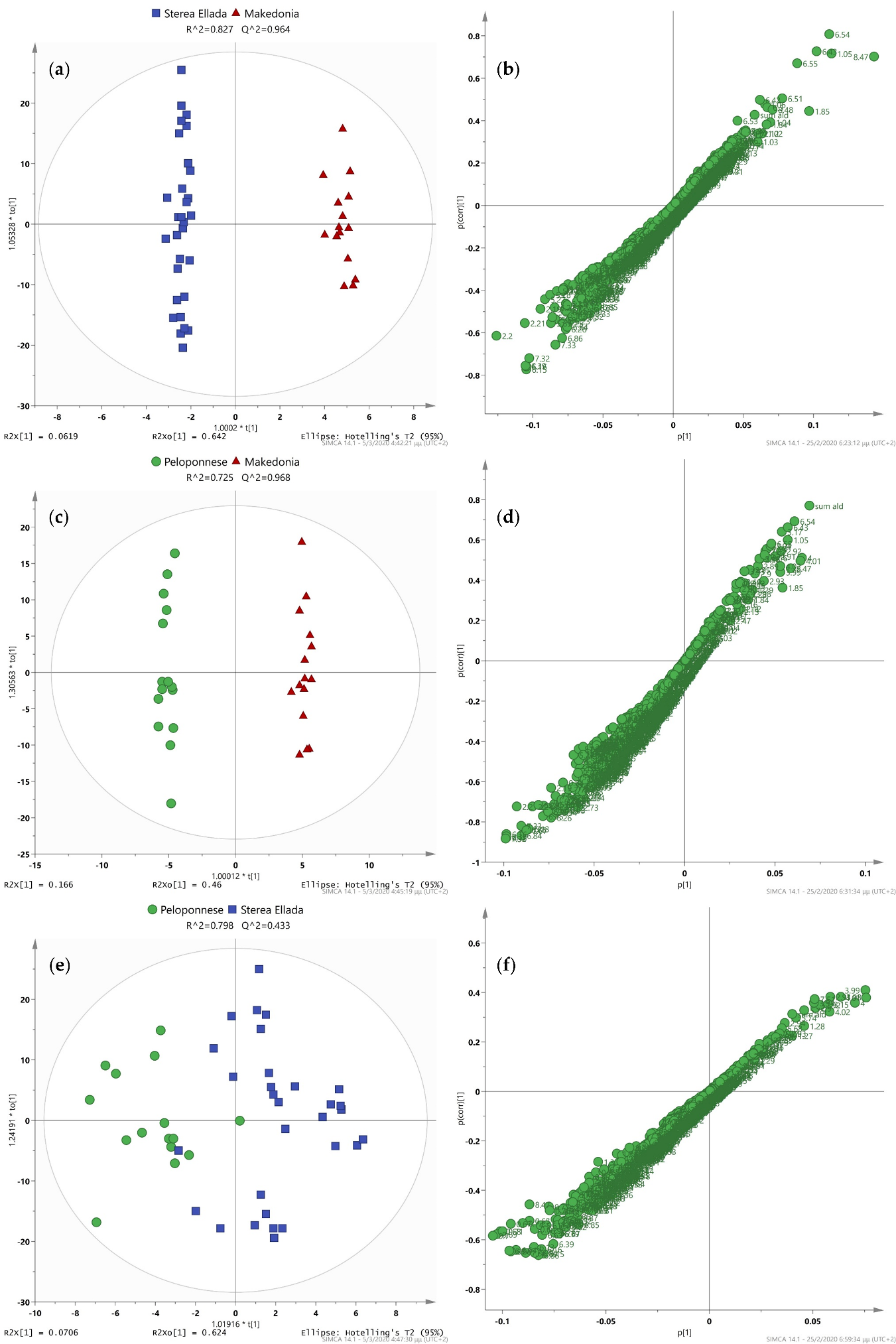


**Figure S2.** OPLS-DA models for the geographical origin parameter with their respective S-plots. (**a**) Sterea Ellada vs Makedonia; (**b**) Respective S-plot; (**c**) Peloponnese vs Makedonia; (**d**) Respective S-plot; (**e**) Peloponnese vs Sterea Ellada; (**f**) Respective S-plot.


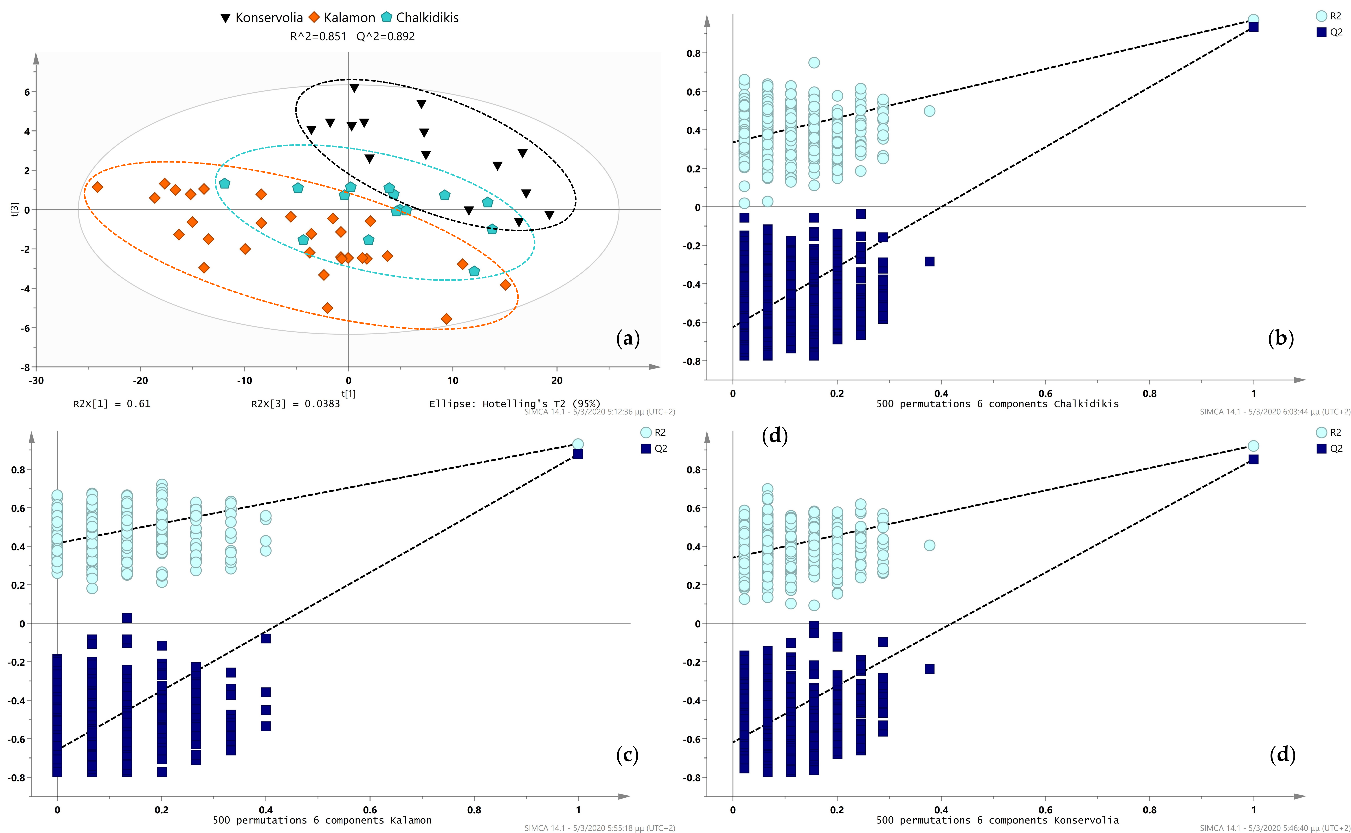


**Figure S3.** (**a**) PLS-DA score plot depicting the formation of three clusters, Konservolia, Kalamon and Chalkidikis; (**b**) Permutation test with 500 permutations performed at the presented PLS-DA model with samples from Chalkidikis variety, verifying the validity of the model.; (**c**) Respective permutation test with samples from Kalamon variety; (**d**) Respective permutation test with samples from Konservolia variety.


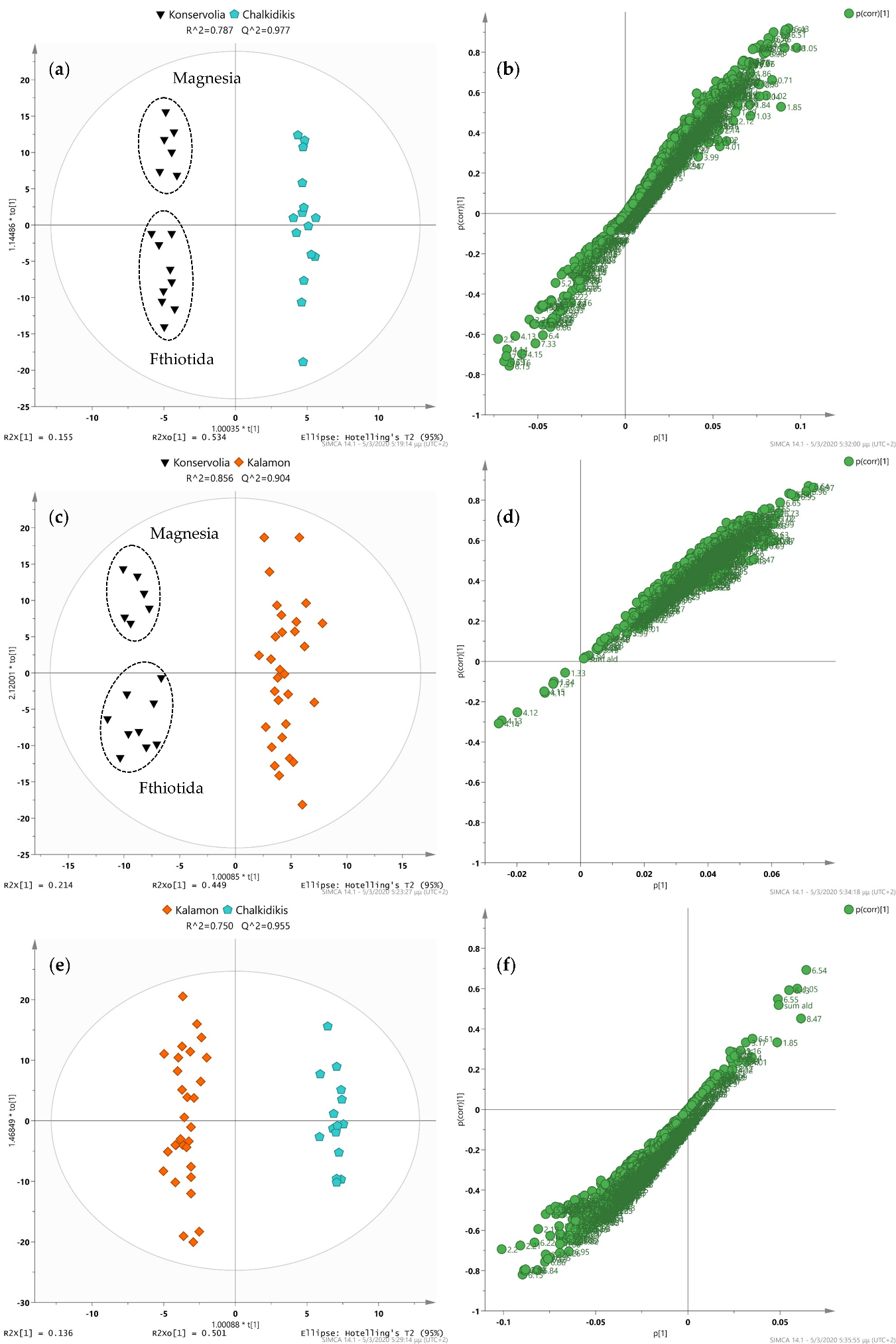


**Figure S4.** OPLS-DA models for the variety parameter with their respective S-plots. (**a**) Konservolia vs Chalkidikis; (**b**) Respective S-plot; (**c**) Konservolia vs Kalamon; (**d**) Respective S-plot; (**e**) Kalamon vs Chalkidikis; (**f**) Respective S-plot.


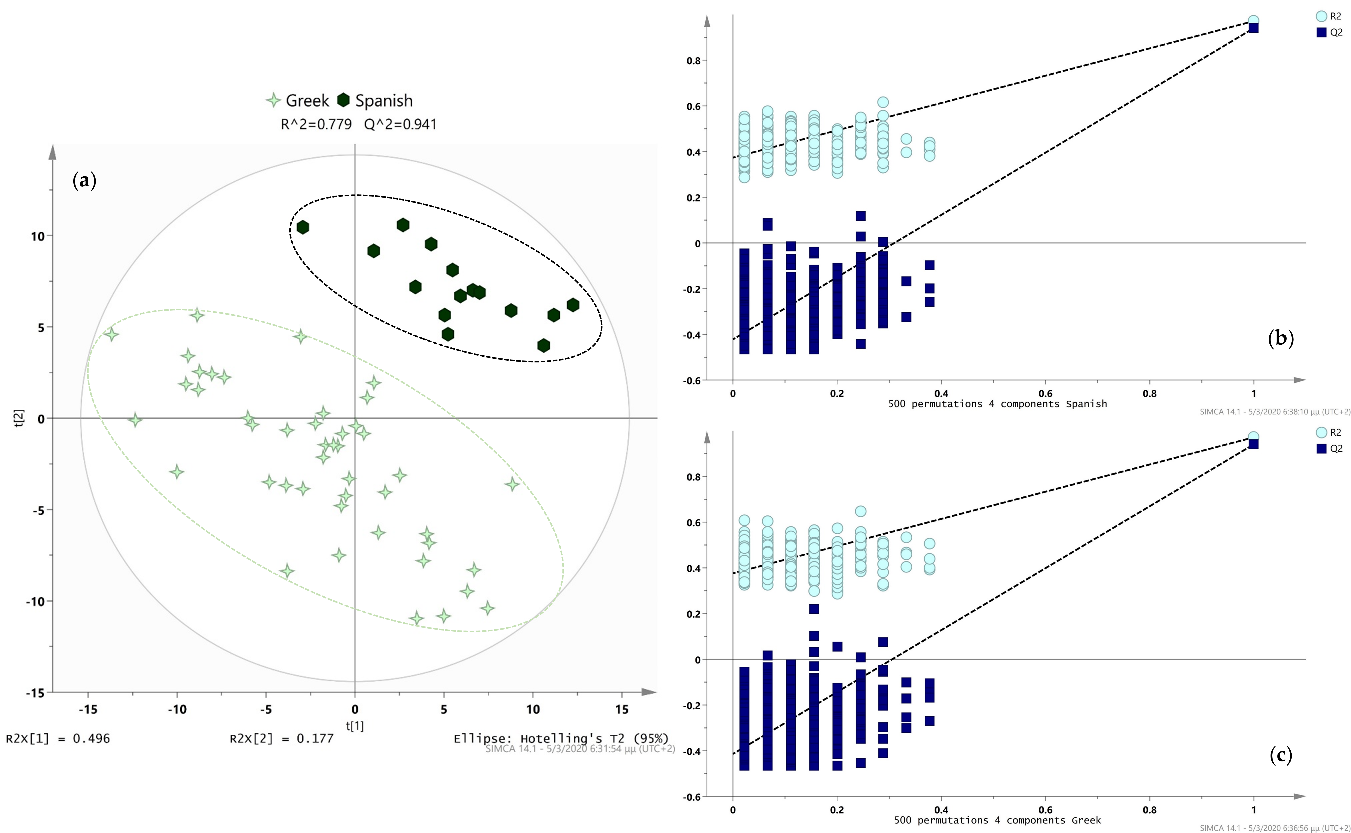


**Figure S5.** (**a**) PLS-DA scores plot with a clear distinction between the samples of Spanish and Greek processing style; (**b**) Permutation test with 500 permutations performed at the presented PLS-DA model with samples that have undergone Greek style processing, verifying the validity of the model.; (**c**) Respective permutation test with samples that have undergone Spanish style processing.


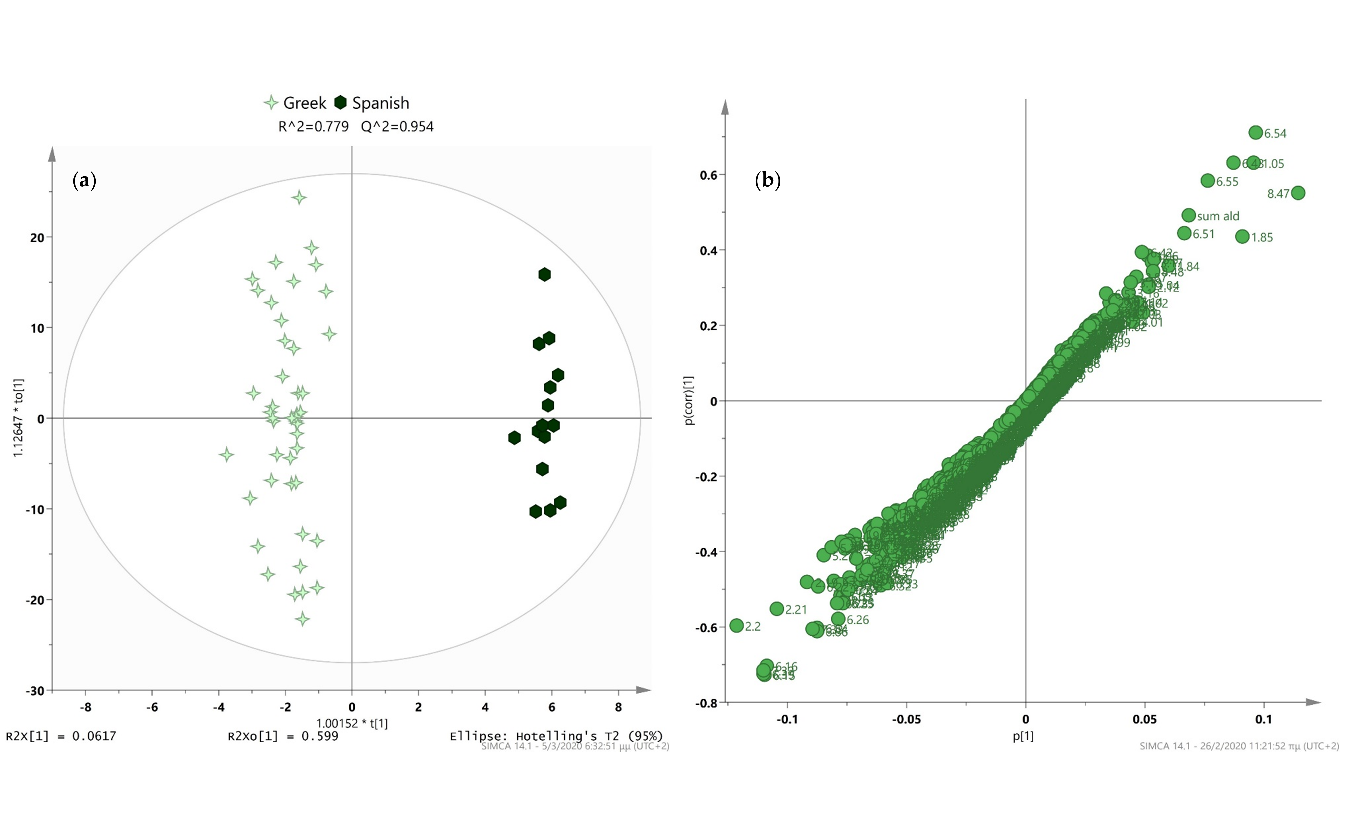


**Figure S6.** (**a**) OPLS-DA model between the samples of Spanish and Greek processing style; (**b**) Respective S-plot.

**Table S2.** VIPs list. Variables in descending order of significance in the formation of the model with the respective identified compounds.

| **Greek vs Spanish** | | | |  | **Sterea Ellada vs Makedonia** | | | |  | **Peloponnese vs Sterea Ellada** | | | |  | **Peloponnese vs Makedonia** | | | |
| --- | --- | --- | --- | --- | --- | --- | --- | --- | --- | --- | --- | --- | --- | --- | --- | --- | --- | --- |
| **No** | **Var ID** | **VIP score** | **Compound** |  | **No** | **Var ID** | **VIP score** | **Compound** |  | **No** | **Var ID** | **VIP score** | **Compound** |  | **No** | **Var ID** | **VIP score** | **Compound** |
| 1 | 2.2 | 2.5550 | TAGs |  | 1 | 8.47 | 2.9328 | FA |  | 1 | 0.7 | 2.1569 | n.i. |  | 1 | 7.32 | 2.1706 | Lut |
| 2 | 8.47 | 2.3774 | FA |  | 2 | 2.2 | 2.6617 | TAGs |  | 2 | 0.69 | 2.0995 | n.i. |  | 2 | 6.15 | 2.1666 | Lut |
| 3 | 7.32 | 2.2569 | Lut |  | 3 | 1.05 | 2.3197 | n.i. |  | 3 | 0.71 | 2.0918 | OA |  | 3 | 6.39 | 2.1645 | Lut |
| 4 | 6.15 | 2.2488 | Lut |  | 4 | 6.54 | 2.2630 | n.i. |  | 4 | 0.68 | 2.0903 | n.i. |  | 4 | 2.2 | 2.0598 | TAGs |
| 5 | 6.39 | 2.2469 | Lut |  | 5 | 6.39 | 2.1535 | Lut |  | 5 | 6.96 | 1.9811 | Tyr |  | 5 | 6.84 | 1.9906 | Lut |
| 6 | 1.05 | 1.9995 | n.i. |  | 6 | 6.15 | 2.1462 | Lut |  | 6 | 6.64 | 1.9383 | Tyr |  | 6 | 7.67 | 1.9398 | Quer |
| 7 | 6.54 | 1.9903 | n.i. |  | 7 | 7.32 | 2.1129 | Lut |  | 7 | 0.65 | 1.8330 | n.i. |  | 7 | 6.13 | 1.9106 | Quer |
| 8 | 5.27 | 1.9619 | TAGs |  | 8 | 6.43 | 2.0967 | n.i. |  | 8 | 7.67 | 1.8261 | Quer |  | 8 | 6.22 | 1.8456 | Ver |
| 9 | 1.85 | 1.9306 | AA |  | 9 | 5.27 | 2.0630 | TAGs |  | 9 | 8.47 | 1.7782 | FA |  | 9 | 6.75 | 1.7565 | n.i. |
| 10 | 6.22 | 1.8908 | Ver |  | 10 | 1.85 | 2.0560 | AA |  | 10 | 6.13 | 1.7657 | Quer |  | 10 | 6.99 | 1.7012 | Ver |
| 11 | 6.84 | 1.8339 | Lut |  | 11 | 5.69 | 1.8720 | n.i. |  | 11 | 4.01 | 1.7457 | LA |  | 11 | 6.26 | 1.6730 | n.i. |
| 12 | 6.43 | 1.8134 | n.i. |  | 12 | 5.73 | 1.8665 | n.i. |  | 12 | 0.64 | 1.7442 | n.i. |  | 12 | 7.54 | 1.6664 | Ver |
| 13 | 1.97 | 1.7812 | TAGs |  | 13 | 6.22 | 1.8368 | Ver |  | 13 | 6.84 | 1.7276 | Lut |  | 13 | 6.68 | 1.6450 | n.i. |
| 14 | 5.69 | 1.6946 | n.i. |  | 14 | 1.97 | 1.8231 | TAGs |  | 14 | 7.33 | 1.7198 | Lut |  | 14 | 6.34 | 1.6142 | Quer |
| 15 | 6.75 | 1.6909 | n.i. |  | 15 | 5.68 | 1.8050 | n.i. |  | 15 | 5.61 | 1.6970 | n.i. |  | 15 | 7.58 | 1.6101 | Quer |
| 16 | 6.26 | 1.6752 | n.i. |  | 16 | 5.74 | 1.6786 | n.i. |  | 16 | 0.73 | 1.6871 | n.i. |  | 16 | 6.72 | 1.6055 | Ver |
| 17 | 6.13 | 1.6541 | Quer |  | 17 | 5.72 | 1.6758 | n.i. |  | 17 | 0.76 | 1.6586 | MA/OA |  | 17 | Ald | 1.5528 | n.i. |
| 18 | 6.24 | 1.6366 | n.i. |  | 18 | 6.86 | 1.6662 | Lut |  | 18 | 6.15 | 1.6441 | Lut |  | 18 | 5.27 | 1.5516 | TAGs |
| 19 | 5.73 | 1.6246 | n.i. |  | 19 | 6.51 | 1.6573 | Ver |  | 19 | 6.78 | 1.5861 | n.i. |  | 19 | 6.82 | 1.5234 | Quer |
| 20 | 1.24 | 1.6205 | TAGs |  | 20 | 6.24 | 1.6533 | n.i. |  | 20 | 6.72 | 1.5845 | Ver |  | 20 | 4.52 | 1.4904 | n.i. |
| 21 | 0.83 | 1.6115 | TAGs |  | 21 | 1.24 | 1.6414 | TAGs |  | 21 | 2.65 | 1.5654 | Tyr |  | 21 | 6.95 | 1.4845 | Tyr |
| 22 | 7.54 | 1.6092 | Ver |  | 22 | 6.26 | 1.6206 | n.i. |  | 22 | 6.39 | 1.5456 | Lut |  | 22 | 1.97 | 1.4717 | TAGS |
| 23 | 7.67 | 1.5975 | Quer |  | 23 | 0.83 | 1.6012 | TAGs |  | 23 | 6.68 | 1.5442 | n.i. |  | 23 | 6.65 | 1.4504 | Tyr |
| 24 | 5.68 | 1.5972 | n.i. |  | 24 | 2 | 1.5666 | TAGs |  | 24 | 6.82 | 1.4906 | Quer |  | 24 | 7.4 | 1.4309 | n.i. |
| 25 | 2.01 | 1.5526 | TAGs |  | 25 | 6.75 | 1.5437 | n.i. |  | 25 | 0.74 | 1.4714 | MA |  | 25 | 4 | 1.4214 | LA |
| 26 | 5.72 | 1.5217 | n.i. |  | 26 | 1.04 | 1.5392 | PA |  | 26 | 1.02 | 1.4297 | Ver |  | 26 | 8.47 | 1.4173 | FA |
| **No** | **Var ID** | **VIP score** | **Compound** |  | **No** | **Var ID** | **VIP score** | **Compound** |  | **No** | **Var ID** | **VIP score** | **Compound** |  | **No** | **Var ID** | **VIP score** | **Compound** |
| 27 | 1.54 | 1.5085 | TAGs |  | 27 | 5.7 | 1.5182 | n.i. |  | 27 | 0.77 | 1.4143 | n.i. |  | 27 | 5.6 | 1.4171 | n.i. |
| 28 | ald | 1.4819 | n.i. |  | 28 | 5.75 | 1.5096 | n.i. |  | 28 | 7.58 | 1.4123 | Quer |  | 28 | 6.17 | 1.4164 | n.i. |
| 29 | 6.68 | 1.4788 | n.i. |  | 29 | 4.14 | 1.5005 | n.i. |  | 29 | 4.5 | 1.3879 | n.i. |  | 29 | 6.9 | 1.4123 | Ver |
| 30 | 6.51 | 1.4728 | Ver |  | 30 | 1.93 | 1.4991 | n.i. |  | 30 | 2.91 | 1.3803 | n.i. |  | 30 | 4.54 | 1.3983 | n.i. |
| 31 | 5.74 | 1.4645 | n.i. |  | 31 | 1.02 | 1.4744 | PA/Ver |  | 31 | 1.3 | 1.3780 | LA |  | 31 | 6.54 | 1.3975 | n.i. |
| 32 | 6.99 | 1.4560 | Ver |  | 32 | 7.54 | 1.4718 | Ver |  | 32 | 6.75 | 1.3719 | n.i. |  | 32 | 4.41 | 1.3754 | n.i. |
| 33 | 1.93 | 1.4408 | n.i. |  | 33 | 1.54 | 1.4626 | TAGs |  | 33 | 0.66 | 1.3694 | n.i. |  | 33 | 2.71 | 1.3631 | Lin |
| 34 | 7.36 | 1.4302 | n.i. |  | 34 | 7.36 | 1.4621 | n.i. |  | 34 | 0.78 | 1.3693 | n.i. |  | 34 | 4.46 | 1.3629 | n.i. |
| 35 | 6.29 | 1.4291 | n.i. |  | 35 | 6.29 | 1.4251 | n.i. |  | 35 | 6.89 | 1.3683 | Ver |  | 35 | 1.05 | 1.3561 | n.i. |
| 36 | 4.41 | 1.3925 | n.i. |  | 36 | 2.12 | 1.3934 | PA |  | 36 | 6.74 | 1.3561 | n.i. |  | 36 | 6.29 | 1.3549 | n.i. |
| 37 | 5.7 | 1.3896 | n.i. |  | 37 | 4.09 | 1.3919 | TAGs |  | 37 | 6.99 | 1.3420 | Ver |  | 37 | 7.37 | 1.3509 | n.i. |
| 38 | 2.71 | 1.3748 | Lin |  | 38 | 6.68 | 1.3803 | n.i. |  | 38 | 7.75 | 1.3300 | n.i. |  | 38 | 5.69 | 1.3482 | n.i. |
| 39 | 5.88 | 1.3564 | n.i. |  | 39 | 5.71 | 1.3638 | n.i. |  | 39 | 1 | 1.3203 | n.i. |  | 39 | 6.37 | 1.3435 | n.i. |
| 40 | 7.4 | 1.3552 | n.i. |  | 40 | 4.41 | 1.3594 | n.i. |  | 40 | 0.79 | 1.3104 | n.i. |  | 40 | 0.83 | 1.3389 | TAGs |
| 41 | 6.34 | 1.3548 | Quer |  | 41 | 1.33 | 1.3414 | LA |  | 41 | 4.52 | 1.3089 | n.i. |  | 41 | 1.85 | 1.3259 | AA |
| 42 | 7.57 | 1.3435 | Quer |  | 42 | 5.88 | 1.3270 | n.i. |  | 42 | 2.92 | 1.3054 | n.i. |  | 42 | 2.01 | 1.3211 | TAGs |
| 43 | 7.37 | 1.3349 | n.i. |  | 43 | 6.13 | 1.3264 | Quer |  | 43 | 1.28 | 1.2823 | TAGs |  | 43 | 4.32 | 1.3209 | Ver |
| 44 | 3.75 | 1.3207 | n.i. |  | 44 | 7.56 | 1.3258 | n.i. |  | 44 | 6.93 | 1.2811 | n.i. |  | 44 | 1.54 | 1.3200 | TAGs |
| 45 | 6.37 | 1.3173 | n.i. |  | 45 | 6.37 | 1.3229 | n.i. |  | 45 | 7.78 | 1.2719 | n.i. |  | 45 | 1.24 | 1.3195 | TAGs |
| 46 | 4.09 | 1.3148 | TAGs |  | 46 | ald | 1.3162 | n.i. |  | 46 | 2.9 | 1.2712 | n.i. |  | 46 | 2.74 | 1.3057 | n.i. |
| 47 | 6.17 | 1.2969 | n.i. |  | 47 | 2.71 | 1.3034 | Lin |  | 47 | 1.74 | 1.2671 | n.i. |  | 47 | 2.92 | 1.3031 | n.i. |
| 48 | 7.38 | 1.2761 | n.i. |  | 48 | 6.33 | 1.2851 | Quer |  | 48 | 4.38 | 1.2647 | n.i. |  | 48 | 6.43 | 1.3023 | n.i. |
| 49 | 6.72 | 1.2730 | Ver |  | 49 | 0.71 | 1.2848 | OA |  | 49 | 6.34 | 1.2295 | Quer |  | 49 | 7.59 | 1.2998 | n.i. |
| 50 | 5.75 | 1.2665 | n.i. |  | 50 | 3.75 | 1.2737 | n.i. |  | 50 | 6.7 | 1.2241 | n.i. |  | 50 | 1.31 | 1.2968 | LA |
| 51 | 3.49 | 1.2551 | n.i. |  | 51 | 7.37 | 1.2678 | n.i. |  | 51 | 3.94 | 1.2234 | n.i. |  | 51 | 4.48 | 1.2861 | n.i. |
| 52 | 4.32 | 1.2484 | Ver |  | 52 | 7.67 | 1.2353 | Quer |  | 52 | 6.22 | 1.2226 | Ver |  | 52 | 0.65 | 1.2728 | n.i. |
| 53 | 2.12 | 1.2419 | PA |  | 53 | 5.67 | 1.2328 | n.i. |  | 53 | 1.66 | 1.2154 | n.i. |  | 53 | 5.88 | 1.2563 | n.i. |
| 54 | 1.31 | 1.2416 | LA |  | 54 | 2.3 | 1.2303 | TAGs |  | 54 | 7.72 | 1.2120 | n.i. |  | 54 | 3.75 | 1.2508 | n.i. |
| 55 | 1.04 | 1.2415 | PA |  | 55 | 6.99 | 1.2174 | Ver |  | 55 | 4.45 | 1.2005 | n.i. |  | 55 | 5.55 | 1.2456 | n.i. |
| **No** | **Var ID** | **VIP score** | **Compound** |  | **No** | **Var ID** | **VIP score** | **Compound** |  | **No** | **Var ID** | **VIP score** | **Compound** |  | **No** | **Var ID** | **VIP score** | **Compound** |
| 56 | 7.61 | 1.2238 | n.i. |  | 56 | 3.49 | 1.2124 | n.i. |  | 56 | 0.8 | 1.1881 | n.i. |  | 56 | 0.73 | 1.2441 | n.i. |
| 57 | 4 | 1.2217 | LA |  | 57 | 6.6 | 1.2026 | HT |  | 57 | 2.45 | 1.1772 | SA |  | 57 | 1.66 | 1.2368 | n.i. |
| 58 | 2.3 | 1.2153 | TAGs |  | 58 | 0.77 | 1.1962 | n.i. |  | 58 | 1.49 | 1.1757 | n.i. |  | 58 | 2.91 | 1.2218 | n.i. |
| 59 | 6.82 | 1.2082 | Quer |  | 59 | 6.17 | 1.1945 | n.i. |  | 59 | 1.75 | 1.1632 | n.i. |  | 59 | 6.49 | 1.2075 | Lut |
| 60 | 4.46 | 1.2036 | n.i. |  | 60 | 7.38 | 1.1928 | n.i. |  | 60 | 6.61 | 1.1539 | HT |  | 60 | 0.69 | 1.1883 | n.i. |
| 61 | 1.02 | 1.2030 | Ver/PA |  | 61 | 7.49 | 1.1840 | n.i. |  | 61 | 1.07 | 1.1535 | n.i. |  | 61 | 1.93 | 1.1790 | n.i. |
| 62 | 5.71 | 1.2004 | n.i. |  | 62 | 7.4 | 1.1833 | n.i. |  | 62 | 6.26 | 1.1499 | n.i. |  | 62 | 3.49 | 1.1757 | n.i. |
| 63 | 2.74 | 1.1875 | n.i. |  | 63 | 4.97 | 1.1832 | n.i. |  | 63 | 1.09 | 1.1476 | OA |  | 63 | 2.93 | 1.1720 | n.i. |
| 64 | 4.52 | 1.1806 | n.i. |  | 64 | 3.64 | 1.1605 | n.i. |  | 64 | 6.49 | 1.1362 | Lut |  | 64 | 5.38 | 1.1618 | n.i. |
| 65 | 0.64 | 1.1784 | n.i. |  | 65 | 2.1 | 1.1510 | n.i. |  | 65 | 1.05 | 1.1335 | n.i. |  | 65 | 5.72 | 1.1389 | n.i. |
| 66 | 7.49 | 1.1721 | n.i. |  | 66 | 2.87 | 1.1464 | n.i. |  | 66 | 5.75 | 1.1323 | n.i. |  | 66 | 2.87 | 1.1386 | n.i. |
| 67 | 2.87 | 1.1574 | n.i. |  | 67 | 6.46 | 1.1409 | HT |  | 67 | 4.54 | 1.1307 | n.i. |  | 67 | 5.91 | 1.1372 | n.i. |
| 68 | 5.38 | 1.1492 | n.i. |  | 68 | 7.61 | 1.1317 | n.i. |  | 68 | 7.07 | 1.1286 | n.i. |  | 68 | 5.1 | 1.1348 | n.i. |
| 69 | 6.9 | 1.1485 | Ver |  | 69 | 4.32 | 1.1229 | Ver |  | 69 | 7.02 | 1.1230 | n.i. |  | 69 | 4.36 | 1.1330 | n.i. |
| 70 | 4.97 | 1.1460 | n.i. |  | 70 | 4.95 | 1.1207 | n.i. |  | 70 | 5.89 | 1.1176 | n.i. |  | 70 | 3.94 | 1.1220 | n.i. |
| 71 | 4.54 | 1.1415 | n.i. |  | 71 | 4.87 | 1.1061 | n.i. |  | 71 | 6.41 | 1.1167 | n.i. |  | 71 | 2.94 | 1.1196 | n.i. |
| 72 | 7.59 | 1.1367 | n.i. |  | 72 | 0.7 | 1.1034 | n.i. |  | 72 | 6.52 | 1.1095 | Ver |  | 72 | 3.79 | 1.0935 | n.i. |
| 73 | 4.39 | 1.1348 | n.i. |  | 73 | 0.68 | 1.1028 | n.i. |  | 73 | 7.59 | 1.1091 | n.i. |  | 73 | 5.86 | 1.0927 | n.i. |
| 74 | 5.67 | 1.1337 | n.i. |  | 74 | 7.57 | 1.0989 | Quer |  | 74 | 7.52 | 1.0819 | Ver |  | 74 | 4.97 | 1.0916 | n.i. |
| 75 | 1.13 | 1.1276 | n.i. |  | 75 | 4 | 1.0951 | LA |  | 75 | 0.94 | 1.0684 | MA |  | 75 | 6.51 | 1.0845 | Ver |
| 76 | 4.44 | 1.1139 | n.i. |  | 76 | 4.86 | 1.0778 | Ver |  | 76 | 0.97 | 1.0589 | n.i. |  | 76 | 4.88 | 1.0817 | n.i. |
| 77 | 4.95 | 1.1021 | n.i. |  | 77 | 4.46 | 1.0757 | n.i. |  | 77 | 6.46 | 1.0584 | HT |  | 77 | 2.88 | 1.0811 | n.i. |
| 78 | 3.64 | 1.0972 | n.i. |  | 78 | 5.02 | 1.0740 | n.i. |  | 78 | 6.17 | 1.0570 | n.i. |  | 78 | 2.89 | 1.0805 | n.i. |
| 79 | 6.05 | 1.0737 | n.i. |  | 79 | 5.38 | 1.0686 | n.i. |  | 79 | 5.42 | 1.0548 | n.i. |  | 79 | 7.49 | 1.0803 | n.i. |
| 80 | 5.02 | 1.0622 | n.i. |  | 80 | 4.39 | 1.0652 | n.i. |  | 80 | 7.4 | 1.0379 | n.i. |  | 80 | 6.92 | 1.0734 | n.i. |
| 81 | 2.93 | 1.0603 | n.i. |  | 81 | 2.74 | 1.0622 | n.i. |  | 81 | 2.93 | 1.0374 | n.i. |  | 81 | 4.09 | 1.0703 | TAGs |
| 82 | 5.37 | 1.0588 | n.i. |  | 82 | 2.45 | 1.0600 | SA |  | 82 | 1.85 | 1.0360 | AA |  | 82 | 4.95 | 1.0639 | n.i. |
| 83 | 4.14 | 1.0511 | n.i. |  | 83 | 4.9 | 1.0497 | n.i. |  | 83 | 2.1 | 1.0345 | n.i. |  | 83 | 2.75 | 1.0526 | n.i. |
| 84 | 5.4 | 1.0372 | n.i. |  | 84 | 6.72 | 1.0448 | Ver |  | 84 | 7.27 | 1.0284 | n.i. |  | 84 | 2.9 | 1.0319 | n.i. |
| 85 | 4.86 | 1.0344 | Ver |  | 85 | 1.13 | 1.0430 | n.i. |  | 85 | 1.1 | 1.0274 | MA/OA |  | 85 | 0.7 | 1.0256 | n.i. |
| **No** | **Var ID** | **VIP score** | **Compound** |  | **No** | **Var ID** | **VIP score** | **Compound** |  | **No** | **Var ID** | **VIP score** | **Compound** |  | **No** | **Var ID** | **VIP score** | **Compound** |
| 86 | 5.6 | 1.0339 | n.i. |  | 86 | 2.93 | 1.0396 | n.i. |  | 86 | 5.71 | 1.0140 | n.i. |  | 86 | 2.65 | 1.0222 | Tyr |
| 87 | 6.65 | 1.0314 | Tyr |  | 87 | 4.93 | 1.0333 | n.i. |  | 87 | 1.78 | 1.0130 | n.i. |  | 87 | 2.3 | 1.0167 | TAGs |
| 88 | 6.08 | 1.0312 | n.i. |  | 88 | 6.06 | 1.0301 | n.i. |  | 88 | 1.54 | 1.0003 | TAGs |  | 88 | 3.51 | 1.0100 | G |
| 89 | 5.87 | 1.0264 | n.i. |  | 89 | 2.6 | 1.0159 | HT |  |  |  |  |  |  |  |  |  |  |
| 90 | 1.67 | 1.0106 | n.i. |  | 90 | 4.44 | 1.0043 | n.i. |  |  |  |  |  |  |  |  |  |  |

**Table S2.** (continued)

| **Kalamon vs Chalkidikis** | | | |  | **Kalamon vs Konservolia** | | | |  | **Chalkidikis vs Konservolia** | | | |
| --- | --- | --- | --- | --- | --- | --- | --- | --- | --- | --- | --- | --- | --- |
| **No** | **Var ID** | **VIP score** | **Compound** |  | **No** | **Var ID** | **VIP score** | **Compound** |  | **No** | **Var ID** | **VIP score** | **Compound** |
| 1 | 2.2 | 2.2297 | TAGs |  | 1 | 6.64 | 1.5885 | Tyr |  | 1 | 8.47 | 3.0005 | FA |
| 2 | 6.15 | 1.9330 | Lut |  | 2 | 6.96 | 1.5748 | Tyr |  | 2 | 1.05 | 2.1221 | n.i. |
| 3 | 7.32 | 1.8961 | Lut |  | 3 | 0.71 | 1.4511 | OA |  | 3 | 1.85 | 2.0346 | AA |
| 4 | 6.39 | 1.8961 | Lut |  | 4 | 0.7 | 1.4381 | n.i. |  | 4 | 6.43 | 1.9858 | n.i. |
| 5 | 5.27 | 1.8806 | TAGs |  | 5 | 0.68 | 1.4195 | n.i. |  | 5 | 6.54 | 1.9611 | n.i. |
| 6 | 6.22 | 1.8697 | Ver |  | 6 | 1.02 | 1.4089 | Ver |  | 6 | 6.51 | 1.9607 | Ver |
| 7 | 6.84 | 1.7719 | Lut |  | 7 | 1.3 | 1.4085 | LA |  | 7 | 0.71 | 1.8385 | OA |
| 8 | 1.97 | 1.7662 | TAGs |  | 8 | 6.99 | 1.4078 | Ver |  | 8 | 6.46 | 1.8025 | HT |
| 9 | 6.13 | 1.6924 | Quer |  | 9 | 6.72 | 1.3985 | Ver |  | 9 | 1.02 | 1.7790 | PA |
| 10 | 7.67 | 1.6651 | Quer |  | 10 | 2.65 | 1.3631 | Tyr |  | 10 | 6.64 | 1.7649 | Tyr |
| 11 | 0.83 | 1.6481 | TAGs |  | 11 | 0.73 | 1.3504 | n.i. |  | 11 | 6.96 | 1.7398 | Tyr |
| 12 | 6.75 | 1.6395 | n.i. |  | 12 | 5.61 | 1.3485 | n.i. |  | 12 | 6.6 | 1.7264 | HT |
| 13 | 1.24 | 1.6370 | TAGs |  | 13 | 0.69 | 1.3451 | n.i. |  | 13 | 2.2 | 1.6892 | TAGs |
| 14 | 7.54 | 1.6130 | Ver |  | 14 | 6.22 | 1.3410 | Ver |  | 14 | 0.7 | 1.6842 | n.i. |
| 15 | 6.99 | 1.5918 | Ver |  | 15 | 0.65 | 1.3316 | n.i. |  | 15 | 0.68 | 1.6727 | n.i. |
| 16 | 1.54 | 1.5744 | TAGs |  | 16 | 8.47 | 1.3240 | FA |  | 16 | 2.6 | 1.6644 | HT |
| 17 | 2.01 | 1.5553 | TAGs |  | 17 | 2.71 | 1.3221 | Lin |  | 17 | 7.78 | 1.5732 | n.i. |
| 18 | 6.26 | 1.5509 | n.i. |  | 18 | 7.59 | 1.3079 | n.i. |  | 18 | 1 | 1.5518 | n.i. |
| 19 | 5.69 | 1.5423 | n.i. |  | 19 | 6.61 | 1.3015 | HT |  | 19 | 6.39 | 1.5481 | Lut |
| **No** | **Var ID** | **VIP score** | **Compound** |  | **No** | **Var ID** | **VIP score** | **Compound** |  | **No** | **Var ID** | **VIP score** | **Compound** |
| 20 | 6.68 | 1.5196 | n.i. |  | 20 | 6.9 | 1.2958 | Ver |  | 20 | 7.32 | 1.5458 | Lut |
| 21 | 2.71 | 1.4892 | Lin |  | 21 | 0.83 | 1.2913 | TAGs |  | 21 | 2.1 | 1.5281 | n.i. |
| 22 | 6.72 | 1.4840 | Ver |  | 22 | 1.54 | 1.2842 | TAGs |  | 22 | 3.61 | 1.5004 | HT/Tyr |
| 23 | 6.24 | 1.4611 | n.i. |  | 23 | 1.97 | 1.2818 | TAGs |  | 23 | 7.27 | 1.4880 | n.i. |
| 24 | 7.58 | 1.4510 | Quer |  | 24 | 7.54 | 1.2722 | Ver |  | 24 | 1.29 | 1.4799 | LA |
| 25 | 6.54 | 1.4346 | n.i. |  | 25 | 5.27 | 1.2721 | TAGs |  | 25 | 2.12 | 1.4766 | PA |
| 26 | 7.4 | 1.4227 | n.i. |  | 26 | 0.66 | 1.2670 | n.i. |  | 26 | 6.15 | 1.4748 | Lut |
| 27 | 5.73 | 1.4219 | n.i. |  | 27 | 2.2 | 1.2636 | TAGs |  | 27 | 2.87 | 1.4715 | n.i. |
| 28 | 6.95 | 1.4148 | Tyr |  | 28 | 1.24 | 1.2549 | TAGs |  | 28 | 0.77 | 1.4647 | n.i. |
| 29 | 1.31 | 1.4030 | LA |  | 29 | 7.67 | 1.2504 | Quer |  | 29 | 1.72 | 1.4532 | n.i. |
| 30 | 3.75 | 1.3989 | n.i. |  | 30 | 6.13 | 1.2498 | Quer |  | 30 | 4 | 1.4377 | LA |
| 31 | 8.47 | 1.3946 | FA |  | 31 | 7.4 | 1.2495 | n.i. |  | 31 | 2.65 | 1.4276 | Tyr |
| 32 | 2.74 | 1.3652 | n.i. |  | 32 | 2.01 | 1.2485 | TAGs |  | 32 | 1.89 | 1.4226 | n.i. |
| 33 | 6.82 | 1.3649 | Quer |  | 33 | 6.46 | 1.2473 | HT |  | 33 | 2.45 | 1.4116 | SA |
| 34 | 6.65 | 1.3564 | Tyr |  | 34 | 7.78 | 1.2411 | n.i. |  | 34 | 0.69 | 1.4089 | n.i. |
| 35 | 1.05 | 1.3505 | n.i. |  | 35 | 1.66 | 1.2235 | n.i. |  | 35 | 7.21 | 1.4068 | n.i. |
| 36 | 3.49 | 1.3380 | n.i. |  | 36 | 0.77 | 1.2205 | n.i. |  | 36 | 7.75 | 1.3831 | n.i. |
| 37 | 7.36 | 1.3377 | n.i. |  | 37 | 2.74 | 1.2170 | n.i. |  | 37 | 6.71 | 1.3700 | Ver |
| 38 | 1.93 | 1.3357 | n.i. |  | 38 | 7.58 | 1.2157 | Quer |  | 38 | 0.97 | 1.3668 | n.i. |
| 39 | 6.9 | 1.3275 | Ver |  | 39 | 0.79 | 1.2135 | n.i. |  | 39 | 2.05 | 1.3495 | n.i. |
| 40 | 4.32 | 1.3205 | Ver |  | 40 | 6.69 | 1.2116 | n.i. |  | 40 | sum ald | 1.3305 | n.i. |
| 41 | 7.38 | 1.3201 | n.i. |  | 41 | 2.6 | 1.2103 | HT |  | 41 | 1.38 | 1.3078 | n.i. |
| 42 | 7.37 | 1.3197 | n.i. |  | 42 | 1 | 1.2086 | n.i. |  | 42 | 0.76 | 1.2827 | MA/OA |
| 43 | 7.59 | 1.2994 | n.i. |  | 43 | 3.61 | 1.2083 | HT/Tyr |  | 43 | 0.79 | 1.2817 | n.i. |
| 44 | 5.88 | 1.2924 | n.i. |  | 44 | 1.18 | 1.1925 | n.i. |  | 44 | 1.48 | 1.2653 | n.i. |
| 45 | 4.09 | 1.2894 | TAGs |  | 45 | 3.62 | 1.1924 | HT/Tyr |  | 45 | 2.07 | 1.2579 | n.i. |
| 46 | 6.29 | 1.2873 | n.i. |  | 46 | 0.74 | 1.1830 | MA |  | 46 | 0.66 | 1.2469 | n.i. |
| 47 | 5.6 | 1.2807 | n.i. |  | 47 | 6.82 | 1.1801 | Quer |  | 47 | 7.55 | 1.2388 | Ver |
| 48 | 2.3 | 1.2706 | TAGs |  | 48 | 6.93 | 1.1785 | n.i. |  | 48 | 6.7 | 1.2221 | n.i. |
| **No** | **Var ID** | **VIP score** | **Compound** |  | **No** | **Var ID** | **VIP score** | **Compound** |  | **No** | **Var ID** | **VIP score** | **Compound** |
| 49 | 6.43 | 1.2641 | n.i. |  | 49 | 0.97 | 1.1731 | n.i. |  | 49 | 5.74 | 1.2214 | n.i. |
| 50 | 6.34 | 1.2586 | Quer |  | 50 | 5.55 | 1.1711 | n.i. |  | 50 | 7.04 | 1.2173 | n.i. |
| 51 | 4.41 | 1.2536 | n.i. |  | 51 | 6.84 | 1.1710 | Lut |  | 51 | 1.8 | 1.2147 | n.i. |
| 52 | 7.61 | 1.2527 | n.i. |  | 52 | 0.76 | 1.1677 | MA/OA |  | 52 | 3.62 | 1.2075 | HT/Tyr |
| 53 | 1.13 | 1.2512 | n.i. |  | 53 | 4.06 | 1.1655 | TAGs |  | 53 | 2.36 | 1.2041 | n.i. |
| 54 | 6.37 | 1.2250 | n.i. |  | 54 | 7.27 | 1.1651 | n.i. |  | 54 | 6.93 | 1.2021 | n.i. |
| 55 | 6.17 | 1.2204 | n.i. |  | 55 | 3.75 | 1.1608 | n.i. |  | 55 | 2.86 | 1.1992 | MA |
| 56 | sum ald | 1.2169 | n.i. |  | 56 | 2.08 | 1.1589 | n.i. |  | 56 | 3 | 1.1984 | n.i. |
| 57 | 4.52 | 1.1984 | n.i. |  | 57 | 1.74 | 1.1527 | n.i. |  | 57 | 0.92 | 1.1925 | n.i. |
| 58 | 1.66 | 1.1821 | n.i. |  | 58 | 4.03 | 1.1445 | LA |  | 58 | 2.69 | 1.1786 | TAGs |
| 59 | 5.55 | 1.1761 | n.i. |  | 59 | 5.13 | 1.1432 | Ver |  | 59 | 2.93 | 1.1687 | n.i. |
| 60 | 4.54 | 1.1733 | n.i. |  | 60 | 7.02 | 1.1369 | n.i. |  | 60 | 3.02 | 1.1678 | n.i. |
| 61 | 1.85 | 1.1698 | AA |  | 61 | 5.15 | 1.1274 | n.i. |  | 61 | 1.9 | 1.1634 | n.i. |
| 62 | 5.02 | 1.1673 | n.i. |  | 62 | 3.49 | 1.1214 | n.i. |  | 62 | 5.27 | 1.1534 | TAGs |
| 63 | 4.97 | 1.1666 | n.i. |  | 63 | 2.75 | 1.1143 | n.i. |  | 63 | 0.91 | 1.1502 | OA |
| 64 | 5.37 | 1.1594 | n.i. |  | 64 | 1.09 | 1.1111 | OA |  | 64 | 7.82 | 1.1478 | n.i. |
| 65 | 4.95 | 1.1502 | n.i. |  | 65 | 3.77 | 1.1084 | n.i. |  | 65 | 0.94 | 1.1419 | MA |
| 66 | 1.18 | 1.1446 | n.i. |  | 66 | 4.32 | 1.1049 | Ver |  | 66 | 4.41 | 1.1364 | n.i. |
| 67 | 7.49 | 1.1410 | n.i. |  | 67 | 5.1 | 1.1033 | n.i. |  | 67 | 1.09 | 1.1194 | OA |
| 68 | 4.88 | 1.1410 | n.i. |  | 68 | 7.72 | 1.1007 | n.i. |  | 68 | 3.04 | 1.1101 | n.i. |
| 69 | 4.39 | 1.1280 | n.i. |  | 69 | 3.58 | 1.1007 | G |  | 69 | 5.17 | 1.1042 | MA/OA |
| 70 | 4.86 | 1.1221 | Ver |  | 70 | 1.89 | 1.0998 | n.i. |  | 70 | 5.18 | 1.1023 | MA/OA |
| 71 | 5.1 | 1.1101 | n.i. |  | 71 | 6.75 | 1.0963 | n.i. |  | 71 | 2.99 | 1.0974 | n.i. |
| 72 | 4.46 | 1.1061 | n.i. |  | 72 | 2.29 | 1.0885 | TAGs |  | 72 | 2.83 | 1.0886 | n.i. |
| 73 | 6.45 | 1.0995 | HT |  | 73 | 1.85 | 1.0884 | AA |  | 73 | 1.79 | 1.0874 | n.i. |
| 74 | 6.92 | 1.0906 | n.i. |  | 74 | 2.45 | 1.0801 | SA |  | 74 | 5.15 | 1.0838 | n.i. |
| 75 | 1.14 | 1.0840 | n.i. |  | 75 | 3.47 | 1.0773 | G |  | 75 | 2.68 | 1.0793 | n.i. |
| 76 | 3.79 | 1.0837 | n.i. |  | 76 | 3.52 | 1.0490 | G |  | 76 | 1.47 | 1.0764 | n.i. |
| 77 | 3.58 | 1.0757 | G |  | 77 | 2.23 | 1.0441 | n.i. |  | 77 | 7.06 | 1.0722 | n.i. |
| **No** | **Var ID** | **VIP score** | **Compound** |  | **No** | **Var ID** | **VIP score** | **Compound** |  | **No** | **Var ID** | **VIP score** | **Compound** |
| 78 | 4.44 | 1.0637 | n.i. |  | 78 | 5.38 | 1.0400 | n.i. |  | 78 | 0.89 | 1.0713 | MA/OA |
| 79 | 0.73 | 1.0613 | n.i. |  | 79 | 2.68 | 1.0393 | n.i. |  | 79 | 2.82 | 1.0671 | n.i. |
| 80 | 3.59 | 1.0537 | HT/Tyr |  | 80 | 1.48 | 1.0337 | n.i. |  | 80 | 0.65 | 1.0625 | n.i. |
| 81 | 4.9 | 1.0398 | n.i. |  | 81 | 5.17 | 1.0336 | MA/OA |  | 81 | 1.27 | 1.0572 | TAGs |
| 82 | 3.06 | 1.0345 | n.i. |  | 82 | 2.41 | 1.0281 | n.i. |  | 82 | 0.95 | 1.0500 | MA |
| 83 | 5.03 | 1.0331 | n.i. |  | 83 | 0.94 | 1.0240 | MA |  | 83 | 0.88 | 1.0447 | MA/OA |
| 84 | 3.51 | 1.0283 | G |  | 84 | 6.51 | 1.0228 | Ver |  | 84 | 1.58 | 1.0416 | TAGs |
| 85 | 4.04 | 1.0189 | LA |  | 85 | 5.16 | 1.0101 | MA/OA |  | 85 | 1.6 | 1.0395 | n.i. |
| 86 | 1.16 | 1.0059 | n.i. |  | 86 | 0.91 | 1.0098 | OA |  | 86 | 6.86 | 1.0328 | Lut |
| 87 | 4.5 | 1.0034 | n.i. |  | 87 | 0.95 | 1.0053 | MA |  |  |  |  |  |
| 88 | 6.49 | 1.0027 | Lut |  |  |  |  |  |  |  |  |  |  |
| 89 | 5.13 | 1.0022 | Ver |  |  |  |  |  |  |  |  |  |  |

Abbreviations: AA: Acetic Acid, FA: Formic Acid, G: Glycerol, HT: Hydroxytyrosol, LA: Lactic Acid, Lin: Linoleic Acid, Lut: Luteolin, MA: Maslinic Acid, n.i.: non-identified, OA: Oleanolic Acid, PA: Propionic Acid, Quer: Quercetin, SA: Succinic Acid, TAGs: Triacylglycerols, Tyr: Tyrosol, Ver: Verbascoside.


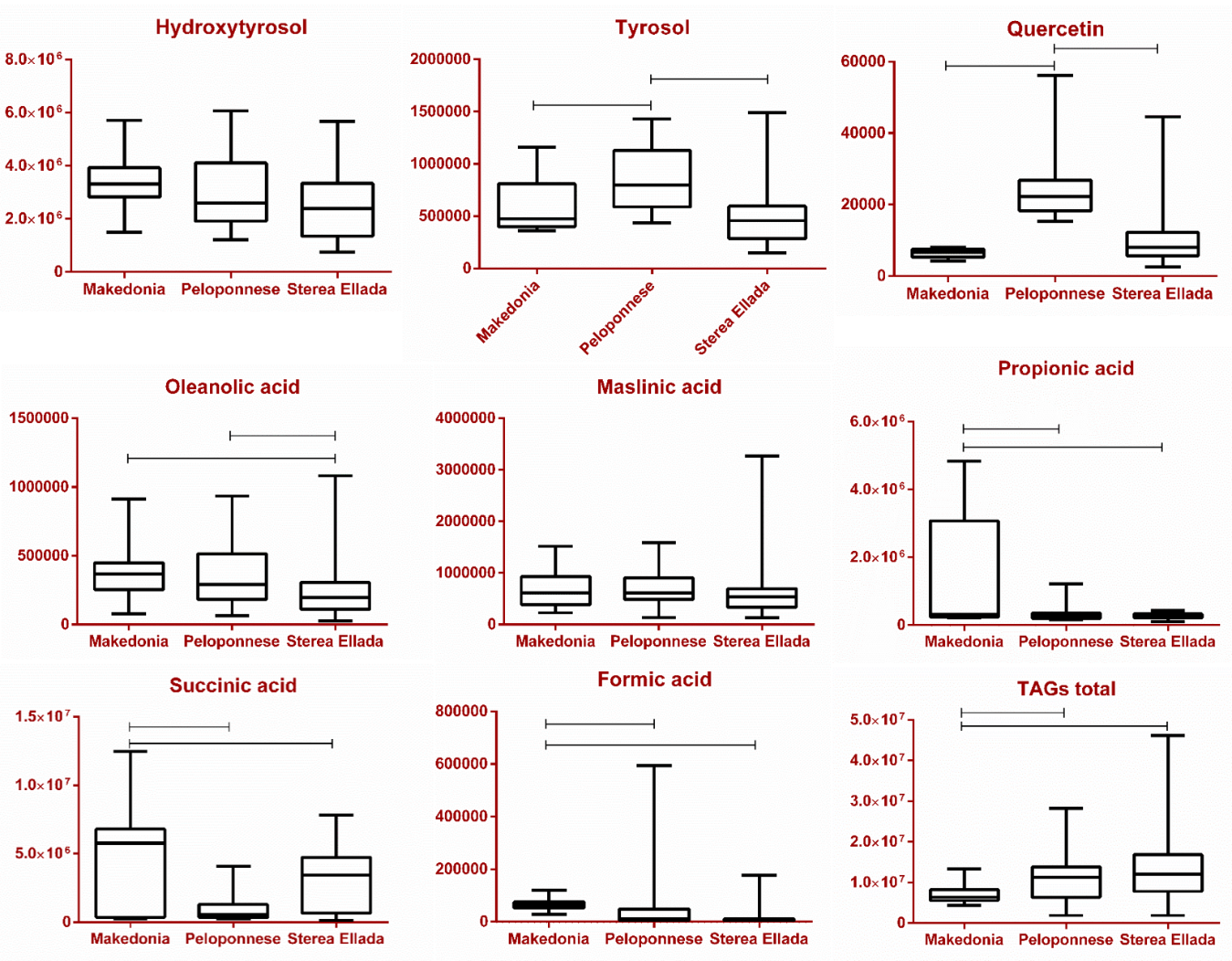


**Figure S7.** Box-plots of the remaining markers in the geographical origin parameter. Depicted are **FA**, **HT**, **Quer**, **MA**, **PA**, **OA**, **Ver**, **Tyr**, total **TAGs** and **SA** (Vertical axis expressed in absolute intensity).


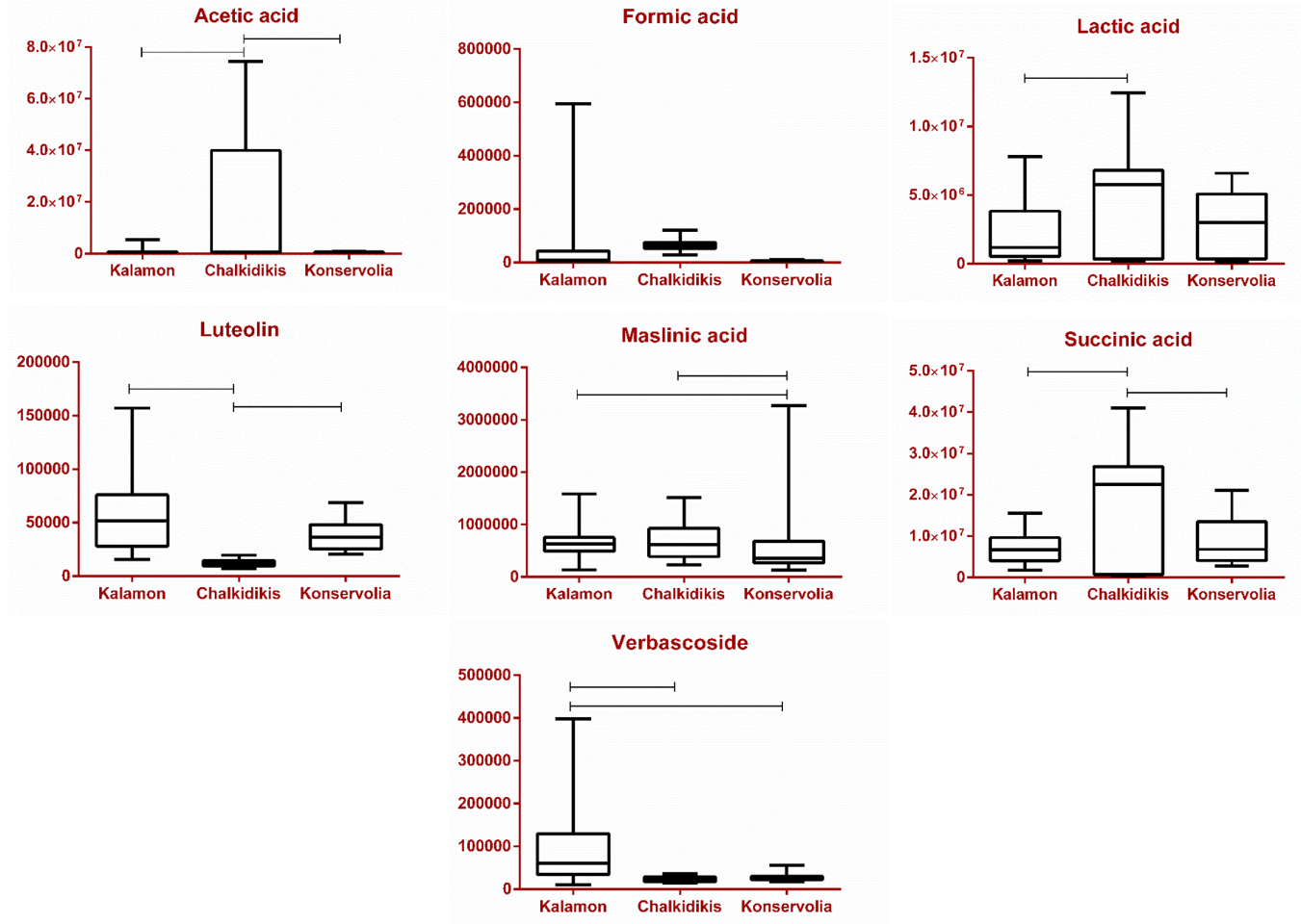


**Figure S8.** Box-plots of the remaining markers in the variety parameter. Depicted are **AA**, **FA**, **LA**, **Lut**, **MA**, **SA** and **Ver** (Vertical axis expressed in absolute intensity).

**Table S3.** T-test applied at the processing method parameter, Greek vs Spanish.

| **Compound** | **p-value** | **Statistically Significant (p<0.05)*** |
| --- | --- | --- |
| **OA** | 0.23322 | No |
| **MA** | 0.87551 | No |
| **TAGs** | 0.00011 | Yes |
| **AA** | 0.02988 | Yes |
| **PA** | 0.01732 | Yes |
| **SA** | 0.00640 | Yes |
| **HT** | 0.06452 | No |
| **Tyr** | 0.82319 | No |
| **LA** | 0.02137 | Yes |
| **Lut** | 0.00000 | Yes |
| **Ver** | 0.00032 | Yes |
| **Quer** | 0.00000 | Yes |
| **FA** | 0.03459 | Yes |

* Statistically significant markers for the discrimination between the two processing methods with p value lower than 0.05 are: **TAGs**, **Quer**, **Lut**, **Ver**, **AA**, **PA**, **SA**, **LA** and **FA**. Values have been rounded up at the fifth decimal digit.
